# Inflammation perturbs hematopoiesis by remodeling specific compartments of the bone marrow niche

**DOI:** 10.1182/blood.2025029513

**Published:** 2026-02-12

**Authors:** James W. Swann, Ruiyuan Zhang, Evgenia V. Verovskaya, Fernando J. Calero-Nieto, Xiaonan Wang, Melissa A. Proven, Hiroyuki Hirakawa, Brian P. Heubel, Peter T. Shyu, X. Edward Guo, Lei Ding, Berthold Göttgens, Emmanuelle Passegué

**Affiliations:** 1 Columbia Stem Cell Initiative, Department of Genetics & Development, Columbia University, New York, NY 10032, USA; 2 Eli and Edythe Broad Center of Regeneration Medicine and Stem Cell Research, Department of Medicine, Division Hematology/Oncology, University of California San Francisco, San Francisco, California 94143, USA; 3 Cambridge Stem Cell Institute, Department of Haematology, Cambridge University, Jeffrey Cheah Biomedical Centre Puddicombe Way, Cambridge CB2 0AW, UK; 4 Columbia Stem Cell Initiative, Department of Rehabilitation & Regenerative Medicine, Department of Microbiology & Immunology, Columbia University, New York, NY 10032, USA; 5 Department of Biomedical Engineering, Columbia University, New York, NY 10032, USA

## Abstract

Hematopoietic stem and progenitor cells (HSPC) are regulated by interactions with stromal cells in the bone marrow (BM) cavity, which can be segregated into two spatially defined central marrow (CM) and endosteal (Endo) compartments. However, the importance of this spatial compartmentalization for BM responses to complex conditions like inflammation remains largely unknown. Here, we extensively validate a combination of scRNA-seq profiling and matching flow cytometry isolation that reproducibly identifies 7 key CM and Endo populations and accurately surveys both niche locations. We demonstrate that inflammatory perturbations exert specific effects on different cellular compartments, with type I interferon responses causing leptin receptor-expressing mesenchymal stromal cells to abandon their normal stromal functions and instead adopt an inflammatory phenotype associated with overproduction of chemokines that modulate local monocyte dynamics in the surrounding microenvironment. Our results provide a comprehensive method for molecular and functional stromal characterization and highlight the importance of altered stomal cell activity in regulating hematopoietic responses to inflammatory challenges.

## Introduction

Hematopoietic stem and progenitor cells (HSPC) respond to demands by increasing production of specific blood lineages^1^, but many hematopoietic responses are also attributable to changes in number and function of both HSPCs and stromal cells located in the bone marrow (BM) cavity. Stromal cells modulate HSPC function in many contexts, including infections^2,3^, aging^4^, and leukemia^5,6^. However, how distinct spatial compartments and cell types of the BM niche respond to different stimuli has received little attention. Gaining insights into these regionalized ecosystems will be instrumental in understanding how specific stromal cell populations can be modulated to mitigate harmful hematopoietic responses.

BM stromal cells like endothelial cells (EC), mesenchymal stromal cells (MSC), and mesenchymal progenitors (MPr) including osteoblast progenitors (OPr), chrondroblast progenitors (CPr), and fibroblast progenitors (FPr)^7,8^, regulate HSPCs through direct contact and by producing soluble ligands. For instance, stromal-derived interleukin (IL)-7 is essential for common lymphoid progenitor (CLP) development^9,10^, whereas production of CXCL12 and stem cell factor (SCF or Kit ligand) by leptin receptor (LepR)-expressing MSCs (MSC-L) and BM ECs retains hematopoietic stem cells (HSC) in the BM niche and enforces HSC quiescence and self-renewal^11–16^. Interactions among HSPCs, stromal cells, and extracellular matrix (ECM) proteins also organize the BM niche into distinct spatial domains with specialized functions^15^.

The BM niche has been characterized by bone section imaging, flow cytometry (FC) of dissociated tissue, and single cell RNA sequencing (scRNA-seq)^17–21^. These complementary approaches describe two principal spatial compartments: the central marrow (CM) niche vascularized by sinusoids, and the endosteal (Endo) niche lining inner bone surfaces and supplied with blood by arterioles^17–20^. Different HSPC populations are enriched in either compartment, with CLPs and some quiescent HSCs with high reconstitution capacity located at the endosteum^12,22,23^, and most HSCs, multipotent progenitors (MPP), granulocyte macrophage progenitors (GMP), and megakaryocytes (Mk) in the CM, sometimes along sinusoidal vessels in production lines^24^. Despite this segregation in cell distribution and function, most investigatory methods do not separate stromal cells originating from spatial compartments, hampering investigations of conditions like inflammation that simultaneously affect both stromal and hematopoietic compartments. This creates a need for a widely applicable technique allowing reliable, prospective isolation of functionally validated stromal populations from both Endo and CM locations to facilitate investigation of perturbations in different spatial compartments of the BM niche.

Here, we describe a refined methodology to separately isolate and profile stromal cells of CM and Endo spatial niches using complementary scRNA-seq profiling and FC. We validate this method against orthogonal studies of the BM niche and establish its broad applicability in profiling stromal populations during inflammatory challenges to understand how changes in specific spatial compartments shape the response of the hematopoietic system. In particular, we identify MSC-L as first responders to type I interferons, licensing them to regulate myeloid responses.

## Methods

### Animal and functional studies

Animal experiments were conducted in accordance with approved Institutional Animal Care and Use Committee protocols and in compliance with all relevant ethical regulations. Mice were injected intraperitoneally with 10 mg/kg polyinosinic:polycytidylic acid (pIC) every 48h for pIC treatment. Other *in vivo* experiments (DGB injection, bone drilling injury model, BM fluid collection), *in vitro* assays (stroma colony, transwell, secretome analyses), and imaging studies were performed as described.

### Combined stromal and hematopoietic cells isolation

From each mouse, 1 femur was used to flush an intact marrow plug and isolate CM stromal populations after two short 10 min digestions with type I collagenase, while all other 7 bones plus the marrow-less femur where crushed to isolate hematopoietic cells and Endo stromal populations after one long 1h digestion with type I collagenase. ACK-treated BM cells were stained for FC analyses of HSPC and mature hematopoietic cells, and Endo and CM cell preparations for FC analyses of various stromal cells.

### Molecular analyses

Single-cell RNA sequencing (scRNA-seq) analyses were performed using either the 10X Genomics Chromium platform and Next GEM Single Cell 3′ Reagent kit version (v.) 3.1 with oligo-hashed TotalSeq B antibodies (BioLegend) or the SMART-Seq Single Cell PLUS kit (Takara Bio), according to the manufacturers’ instructions. Libraries were sequenced on an Illumina NovaSeq 5000 (10X) or Illumina NextSeq 500/550 instrument (SMART-seq). After quality control filtering, data were integrated and analyzed using Seurat v.5 in R. Novel datasets were deposited in the Gene Expression Omnibus (GSE276784).

Detailed methods are described in the Supplemental Information.

## Results

### Profiling BM spatial compartments at single cell resolution

**Figure 1:**
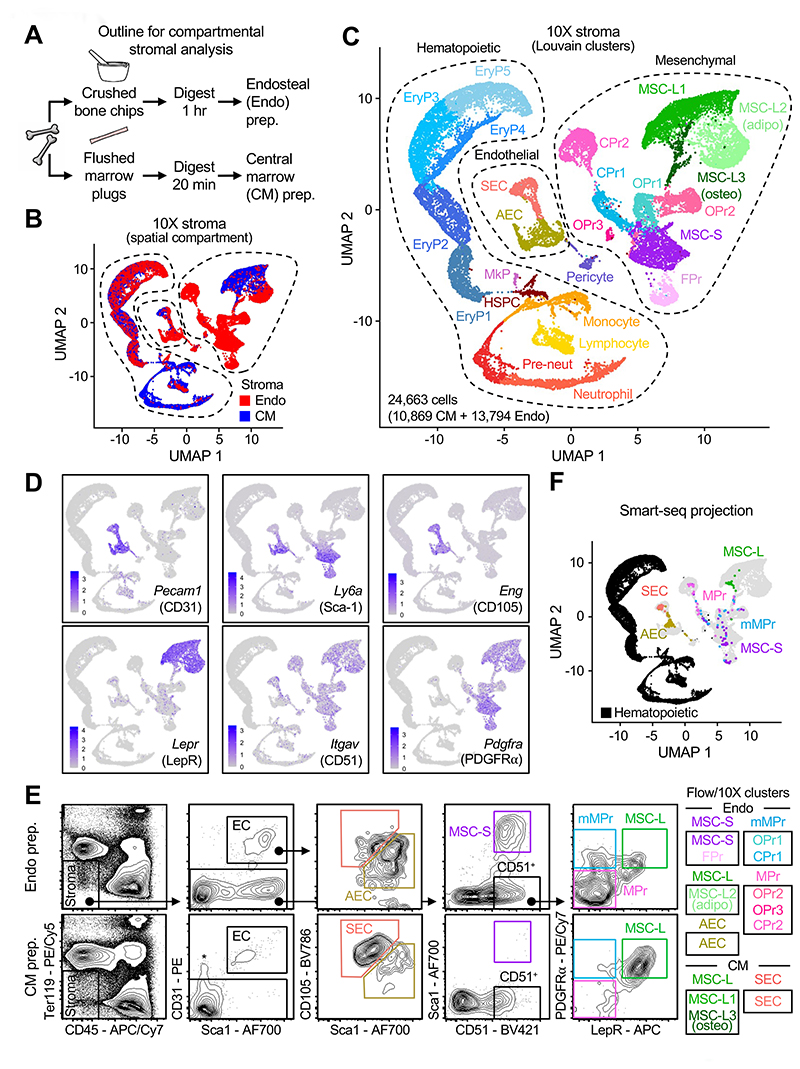
Stromal cells are arranged in two distinct spatial compartments. **A**, Isolation strategy for stromal cells in the central marrow (CM) and endosteal (Endo) fractions. **B**, Uniform manifold and approximation projection (UMAP) of integrated 10X single cell RNA sequencing (scRNA-seq) stroma datasets derived from n = 3 independent experiments profiling CM (blue) and Endo (red) fractions. **C**, UMAP showing Louvain clusters with the numbers of analyzed cells, separation of mesenchymal and endothelial cells from hematopoietic contaminants, and cluster nomenclature. AEC: arterial endothelial cells, SEC: sinusoidal endothelial cell, MSC-S: Sca-1^+^ mesenchymal stromal cell (MSC), OPr: osteoblast progenitor, CPr: chondroblast progenitor, FPr: fibroblast progenitor, MSC-L: leptin receptor (LepR)^+^ MSC (adipo: adipodegnic, osteo: osteogenic), EryP: erythroid progenitor, MkP: megakaryocyte progenitor, HSPC: hematopoietic stem and progenitor cells, Pre-neut, pre-neutrophil. **D**, Feature plots showing expression of indicated genes in the 10X stroma dataset. **E**, Representative flow cytometry plots showing gating of key stromal populations in Endo and CM preparations (prep.) with flow/10X cluster correspondence. (m)MPr: (multipotent) mesenchymal progenitor. * denote contamination by EryP expressing low levels of CD31 marker. **F**, Projection of Smart-seq stromal cell transcriptomes onto the 10X stroma dataset.

We refined a method to isolate separate BM niche compartments from the same mouse by enzymatic digestion of a single intact marrow plug flushed from a femur to obtain CM populations, and bone chips collected from eight bones (1 intact femur + 1 marrow-less femur, 2 tibiae, 2 humeri, 2 hemipelves) crushed together and washed of non-adherent BM cells to obtain Endo populations (Figure 1A). Our goal in pooling different bones was to provide a global view of the Endo niche with enough recoverable cells for molecular and functional studies. Profiling of HSPCs remaining at the endosteum after flushing the marrow plug and in the plug itself confirmed the importance of spatial separation, with previously reported enrichment of HSCs and Mk progenitors (MkP) in CM, and CLPs in the Endo fraction^12,24^ (Supplemental Figures 1A-C). To identify which stromal cells were present at each location, we performed scRNA-seq on Ter119^-^/CD45^-^ CM and Endo cells from young wild type (WT) C57Bl/6 mice in three independent experiments to establish a stroma map (Supplemental Figures 2A-C). Analysis of the integrated dataset revealed little overlap of cells originating from either CM or Endo in clusters (Figure 1B; Supplemental Figure 2B). Significant and variable contamination by hematopoietic cells was also observed across experiments, despite similar initial sorting gates (Figure 1C; Supplemental Figures 2B,D,E; Supplemental Table 1), with both CM and Endo fractions containing CD45^+^ leukocytes and CD45^-^ erythroid progenitors (EryP) that have been described as contaminants of stromal preparations^25^ (Supplemental Figure 2E). In this context, red blood cell lysis was unevenly effective in depleting EryP in our preparations and published results (Supplemental Figure 3A). Despite variations in contaminating hematopoietic cells, recovery of stromal cells was consistent in our three datasets, with all major cell clusters represented across replicates (Supplemental Figure 2B). This establishes the reproductibility of our method to isolate stromal cells from different spatial BM niche locations.

### Spatial compartmentalization of the BM niche resolved by flow cytometry

We next investigated the identity of clusters in our stroma map (Figure 1C). Among EC clusters, we annotated a group of arteriolar endothelial cells (AEC) of almost exclusively Endo origin (96%) and a group of sinusoidal endothelial cells (SEC) of mixed origin (59% CM, 41% Endo) (Supplemental Table 1). Gene expression showed broad expression of *Pecam1* (CD31), with opposite expression patterns for *Ly6a* (Sca-1) and *Eng* (CD105) in AECs vs. SECs (Figure 1D, Supplemental Figure 3B). This was consistent with previously described FC analyses of these two endothelial populations in the Ter119^-^/CD45^-^ stromal fraction^4,26^, with AECs identified as CD31^+^/Sca-1^bright^/CD105^low^ cells found preferentially in the Endo fraction (85.9 ±1.6% of total ECs), and SECs as CD31^+^/Sca-1^low^/CD105^bright^ cells enriched in the CM preparation (70.7 ±7.2% of total ECs) (Figure 1E; Supplemental Figures 3C-E). We also performed scRNA-seq on AECs and SECs isolated from both CM and Endo preparations and projected these FC-purified populations onto our stroma map (Supplemental Figure 4A). Interestingly, while Endo SECs were indistinguishable from CM SECs, the small numbers of CM AECs were rather distinct from Endo AECs and displayed higher *Emcn* expression (Supplemental Figure 4B), likely representing the Endomucin high (Emcn^hi^) type-H vessels described by imaging^27^.

Among mesenchymal cells, we annotated 3 clusters of MSC-Ls of mixed origin, with MSC-L1 and MSC-L3 composed predominantly of CM cells (88% and 67%, respectively) and MSC-L2 mostly of Endo cells (88%) (Figures 1B,C; Supplemental Table 1). Gene expression analyses showed expression of *Cxcl12* (CXCL12), *Itgav* (CD51), and *Pdgfra* (PDGFR-α) across all 3 clusters, but suggested a more committed adipogenic fate in MSC-L2 with higher *Apoe* and *Adipoq* expression, and a strong bias towards osteogenic fate in MSC-L3 as shown by *Alpl* and *Bglap* expression (Figure 1D; Supplemental Figure 4C). This was consistent with our FC isolation of MSC-Ls as Sca-1^-^/CD51^+^/PDGFRα^+^/LepR^+^ cells in the CD31^-^/Ter119^-^/CD45^-^ fraction at both locations, with Endo MSC-Ls representing a minority of Endo mesenchymal cells (2.4 ± 1.0% of CD51^+^ cells) and CM MSC-Ls forming the majority of CM mesenchymal cells (88.1 ± 4.3% of CD51^+^ cells) (Figure 1E; Supplemental Figure 3C). This distribution was further validated by projection of scRNA-seq of FC-purified MSC-Ls isolated from both CM and Endo preparations onto our stroma map, which confirmed that MSC-L2 was primarily composed of Endo MSC-Ls, whereas MSC-L1 derived exclusively from CM MSC-Ls (Supplemental Figure 4A). We also annotated a large mesenchymal group of 7 *Itgav*-expressing clusters of exclusive Endo origin. Co-expression of *Ly6a* and *Pdgfra* identified Sca-1-expressing MSCs (MSC-S), which closely resemble previously defined PαS cells^26,28^, and a further group of fibroblast progenitors (FPr) expressing *Sema3c* (Supplemental Figure 4D; Supplemental Table 1). Additional populations were separated into *Pdgfra*^*+*^ multipotent mesenchymal progenitors (mMPr) and more committed *Pdgfra*^*-*^ mesenchymal progenitors (MPr) with either chondroblastic (CPr1/2) or osteoblastic (OPr1/2/3) bias (Figure 1D; Supplemental Figure 4D; Supplemental Table 1). This again was consistent with our FC isolation of MSC-S (including FPr) as Sca-1^+^/CD51^+^/PDGFRα^+^/LepR^-^ cells, mMPr (representing OPr1/CPr1) as Sca-1^-^/CD51^+^/PDGFRα^+^/LepR^lo^ cells, and MPr (covering OPr2/OPr3/CPr2) as Sca-1^-^/CD51^+^/PDGFRα^−^/LepR^-^ cells in the non-endothelial CD31^-^/Ter119^-^/CD45^-^ fraction of the Endo preparation (13.6 ± 8.6% of endosteal CD51^+^ cells for MSC-S, 4.1 ± 1.8% for mMPr, and 62.3 ± 12.8% for MPr) (Figure 1E; Supplemental Figures 3C). In contrast, the CD31^-^/Ter119^-^/CD45^-^ fraction of the CM preparation was devoid of MSC-S and only had small numbers of (m)MPrs (5.0 ± 2.7% of CM CD51^+^ cells for mMPr, and 5.7 ± 3.3% for MPr). Finally, we found that isolation of mesenchymal cells critically depends on enzymatic tissue digestion, and that using Collagenase I by itself provided excellent release of MSC-L from the marrow plug and acceptable recovery of other stromal cells, though other described digestion protocols achieved better recovery of endothelial and endosteal mesenchymal cells^18,19^ (Supplemental Figure 4E). Taken together, these results indicate that the entire endothelial and mesenchymal compartments can be profiled by FC, with added molecular resolution of population heterogeneity provided by scRNA-seq analyses.

To validate the applicability of our FC scheme, we projected our published plate-based Smart-seq scRNA-seq analyses of the main Endo and CM populations^4^ onto our stroma map (Figure 1F; Supplemental Figures 5A,B; Supplemental Table 2). This confirmed representation of most stromal cell types in our dataset, with pseudobulk gene expression profiles for each cluster correlating strongly with related populations defined by FC (Supplemental Figure 5C). We also projected our Smart-seq profiled populations onto three independent datasets that used different approaches for stromal cell isolation^17–19^, finding in each case that most major stromal populations were captured, albeit with variation in naming conventions (Supplemental Figures 6A,B). To evaluate the conservation of cell types across species, we also compared gene expression profiles from either Smart-seq or 10X stroma map with a human atlas of BM stomal cell types^29^, finding that murine MSC-L strongly resemble human THY^+^ and Adipo-MSC that also express LepR, whereas MSC-S and their endosteal progeny were most similar to Fibro-MSC, which are also biased towards fibroblastic output and enriched in endosteal regions (Supplemental Figures 7A,B). Collectively, these analyses demonstrate that a simple FC method can be used to quantify and prospectively isolate the main stromal cell populations that are spatially segregated into the CM and Endo compartments of the murine BM niche, and which share important features with human BM niche organization.

### Endothelial cells form two vascular niches with distinct functional properties

To characterize FC-isolated CM SECs and Endo AECs further, we first compared their gene expression profile obtained from our published Smart-seq^4^ (Supplemental Table 2). Numerous differentially regulated genes (DEG) were identified, including several markers used previously to distinguish arterial and sinusoidal ECs^17–19^ (Supplemental Figures 8A,B). Geneset enrichment analysis (GSEA) of genes upregulated in SECs revealed enrichment for VEGF signaling (Supplemental Figure 8C), which we confirmed by FC analyses of *Vegfr3-Yfp* mice^30^ (Supplemental Figure 8D). We also found significant enrichment for pathways associated with leukocyte interaction in SECs, with higher expression of selectins and integrins (Supplemental Figure 8E), consistent with sinusoids being interchange sites between BM and blood^31^. In agreement with previous work^32^, we showed that Endomucin, which regulates endothelial:leukocyte interactions and VEGF signaling^33^, was expressed by all SECs and to a much higher extent than AECs (Supplemental Figure 8F). Finally, SECs were enriched for pathways related to vascular permeability and were significantly more permeable to fluorescent dragon green beads (DGB) injected intravenously (Supplemental Figures 8G,H). Conversely, AECs were enriched for DEG signatures related to endothelial integrity (Supplemental Figure 9A), with a subpopulation expressing the gap junction protein connexin 40 (CX40) encoded by *Gja5*, which we confirmed by FC using *Cx40-Gfp* mice^34^ (Supplemental Figure 9B). AECs were also enriched for Notch pathway genes, which we confirmed by FC using *Hes1-Gfp* mice^35^ (Supplemental Figures 9C,D). Collectively, these investigations provide extensive validation that FC-isolated SECs and AECs correspond phenotypically and functionally with similar arterial and sinusoidal EC populations described in previous studies. However, Sca-1 expression, a key identifying marker of AECs, is notoriously labile during inflammation and is expressed negligibly in mice with the Ly6a1 haplotype^36^. We therefore searched for other cell surface markers that distinguish AECs from SECs, finding that CD34 was expressed at significantly higher levels in AECs (Supplemental Figures 9E,F). Accordingly, CD34 effectively distinguished AECs and SECs in Ly6a1 BALB/C mice, which do not express Sca-1 on AECs (Supplemental Figure 9G). Addition of CD34 therefore extends the applicability of our FC scheme to identify AECs and SECs across mouse strains and experimental conditions.

### Mesenchymal lineages are developmentally and spatially segregated in the BM niche

To characterize FC-defined mesenchymal populations, we isolated each major Endo (MSC-S, mMPr, and MPr) and CM (MSC-L) population for *ex vivo* differentiation assays. Although all cell types could generate fibroblast colony forming units (CFU-F) (Supplemental Figure 10A), these were observed much more frequently from Endo populations according to their differentiation gradient (MSC-S > mMPr > MPr) than from CM MSC-Ls, which produced CFU-F infrequently under hypoxic conditions (5% O_2_) and with a ROCK kinase inhibitor (iROCK)^37,38^ (Supplemental Figure 10B). Upon osteoblastic differentiation, MSC-Ls formed the largest alkaline phosphatase (ALP) or von Kossa positive colonies compared to all Endo populations, which in terms of frequency were equivalent to MSC-Ls but formed smaller colonies (Supplemental Figures 10B,D). This confirms the potent bone-forming potential of MSC-Ls, which maintain bone in adult mice and repair fractures^39^, and illustrates the osteogenic potential of MSC-Ss and their (m)MPr derivatives, as demonstrated previously by transplantation^40^. Interesingly, the rare endosteal MSC-L population displayed more vigorous CFU-F and CFU-ALP colony formation than CM MSC-Ls (Supplemental Figure 10E-G), suggesting these subpopulations have distinct functional attributes according to their spatial location. Altogether, these functional results confirm the multipotent nature of MSC-S and MSC-L, yet with non-overlapping biases towards distinct lineages.

**Figure 2:**
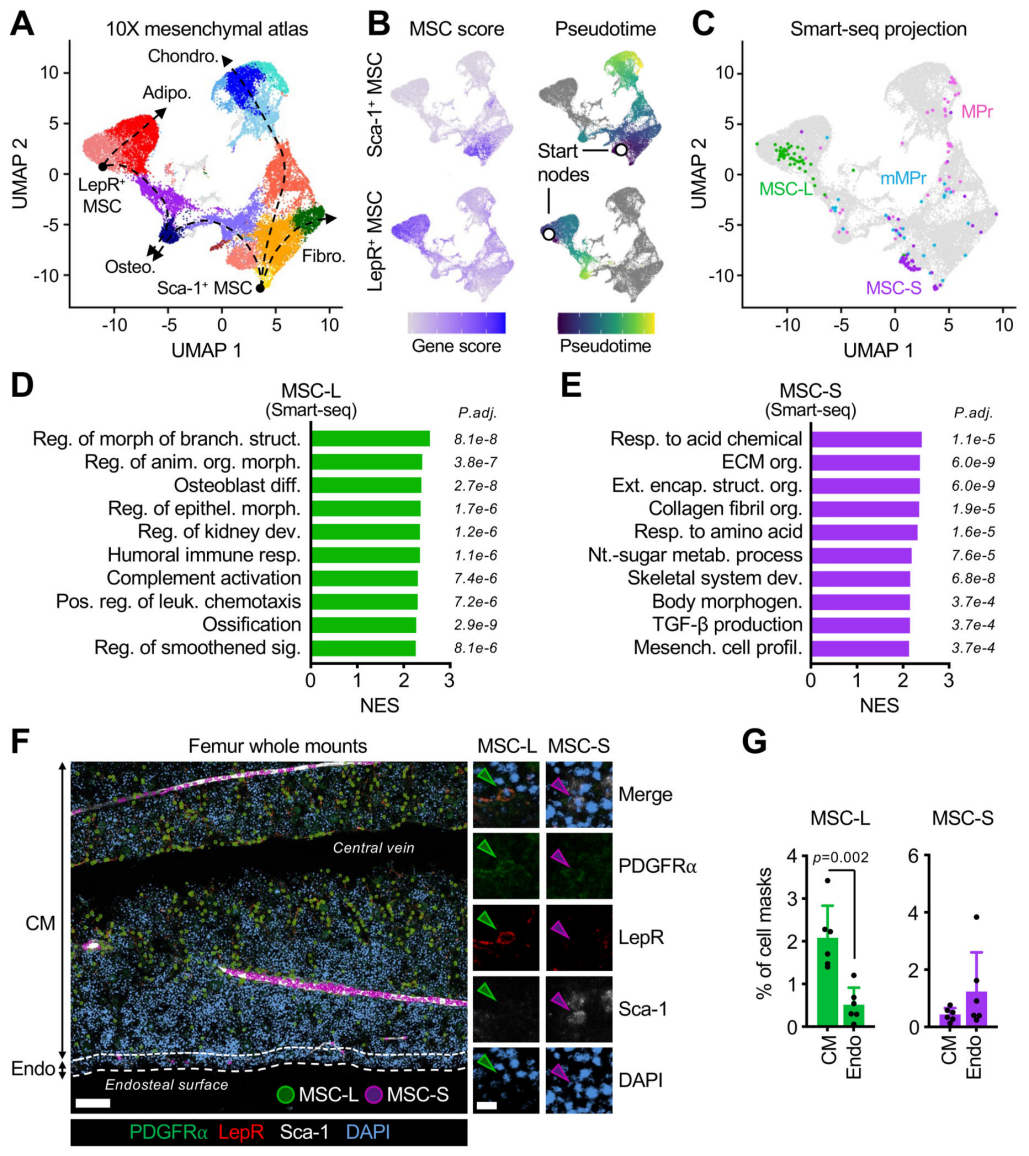
Mesenchymal cells have two distinct origins in the BM niche. **A**, UMAP of a 10X mesenchymal atlas created by integrating n = 3 published 10X scRNA-seq datasets of BM mesenchymal cells^17,18,41^, with major lineage branches indicated. Chrondo.: chondroblastic, Adipo.: adipocytic, Osteo.: osteoblastic, Fibro.: fibroblastic. **B**, Expression of gene signatures associated with Sca-1^+^ or LepR^+^ MSCs (left) and pseudotime calculated with Monocle3 using start nodes corresponding to the highest MSC gene scores (right). **C**, Projection of Smart-seq scRNA-seq transcriptomes onto the 10X mesenchymal atlas. **D-E**, GSEA of selectively enriched GO biological pathways in (D) MSC-L and (E) MSC-S. **F**, Representative confocal image of femur whole mount preparation (scale bar: 100 μm) showing cell masks for MSC-L (green) and MSC-S (purple). **G**, Bar charts showing frequency of MSC-L and MSC-S within central marrow (CM) and endosteal (Endo) bone regions as a proportion of total number of segmented cells. Data are means ± S.D. with points showing values for individual mice. *P. values*, derived from permutation tests (D,E) or Welch’s t tests (G).

Although informative, *ex vivo* assays only define the differentiation potential of cell populations rather than their lineage commitment *in situ*. We therefore cross-referenced our findings with developmental profiles of BM mesenchymal cells obtained by integrating data from three independent published scRNA-seq studies^17,18,41^ to create a mesenchymal cell state atlas (Figure 2A; Supplemental Figures 11A-C). Importantly, integrating datasets with cell preparation differences maximized cell type coverage and provided greater resolution than previous studies^42^ (Supplemental Figure 11D). We used published gene signatures to annotate two sources of BM mesenchymal cells^28,42^: Sca-1^+^ MSCs and LepR^+^ MSCs (Figure 2B; Supplemental Table 3). Using these populations as starting nodes for pseudotime analysis, we inferred that Sca-1^+^ MSCs generated fibroblasts, chondroblasts, and osteoblasts, whereas LepR^+^ MSCs were upstream of osteoblasts and adipocytes (Figure 2B). Importantly, projection of our stroma map onto this mesenchymal atlas confirmed that LepR^+^ MSCs and their progeny were present in both CM and Endo compartments, whereas Sca-1^+^ MSCs and their downstream lineages were confined to the Endo compartment (Supplemental Figure 11E). We also showed strong overlap between the transcriptomes of Sca-1^+^ MSCs and LepR^+^ MSCs with our FC-defined MSC-Ss and MSC-Ls, respectively (Figure 2C). In agreement with their proposed potentiality, we found significant enrichment for GO pathways related to bone formation and mesenchymal development in MSC-Ls, and to fibroblast differentiation and ECM production in MSC-Ss (Figures 2D,E). Our annotation of these two distinct sources of BM mesenchymal cells largely aligns with prior observations. Although both MSC-S and MSC-L cells expressed *Prx1* consistent with their mesenchymal origins^43^ (Supplemental Figure 12), only MSC-Ls overlapped with Nestin-GFP^+^ cells by FC^44^ (Supplemental Figure 12) and correlated with the transcriptomes of cells expressing CXCL12 or Kit ligand^11,13^ (Supplemental Figure 13A). Conversely, MSC-Ss had a similar transcriptome to PαS cells^26^ and expressed both NG2 and CD34, consistent with previous descriptions of periarteriolar and endosteal progenitors with restricted adipose potential^40,45,46^ (Supplemental Figures 12; 13A). These associations were also consistent with the spatial location of MSC-Ss, which were more enriched along the endosteal surface in whole mount imaging (Figure 2F,G; Supplemental Figure 13B), which we defined as a zone of ~15 μm from the inner cortical surface based on imaging femurs from which the marrow plug had been flushed (Supplemental Figure 13C). Conversely, MSC-Ls were significantly more abundant in the CM, as indicated by our FC analyses (Figures 2F,G). Contrary to a previous report suggesting that Gremlin-1 (*Grem1*) identifies a progenitor population with bone and cartilage potential^47^, we found poor expression of *Grem1* in MSC-Ss, with the transcriptome of Grem1^+^ cells correlating more closely with MPrs, in which expression of the osteoblast transcription factor (TF) Osterix (Osx) was also observed^48^ (Supplemental Figures 12; 13A,D). Collectively, these data combined with our functional assays suggest two distinct sources of bone-forming mesenchymal cells in different spatial compartments of the BM niche, which can be resolved and prospectively isolated based on differential Sca-1 and LepR expression.

**Figure 3:**
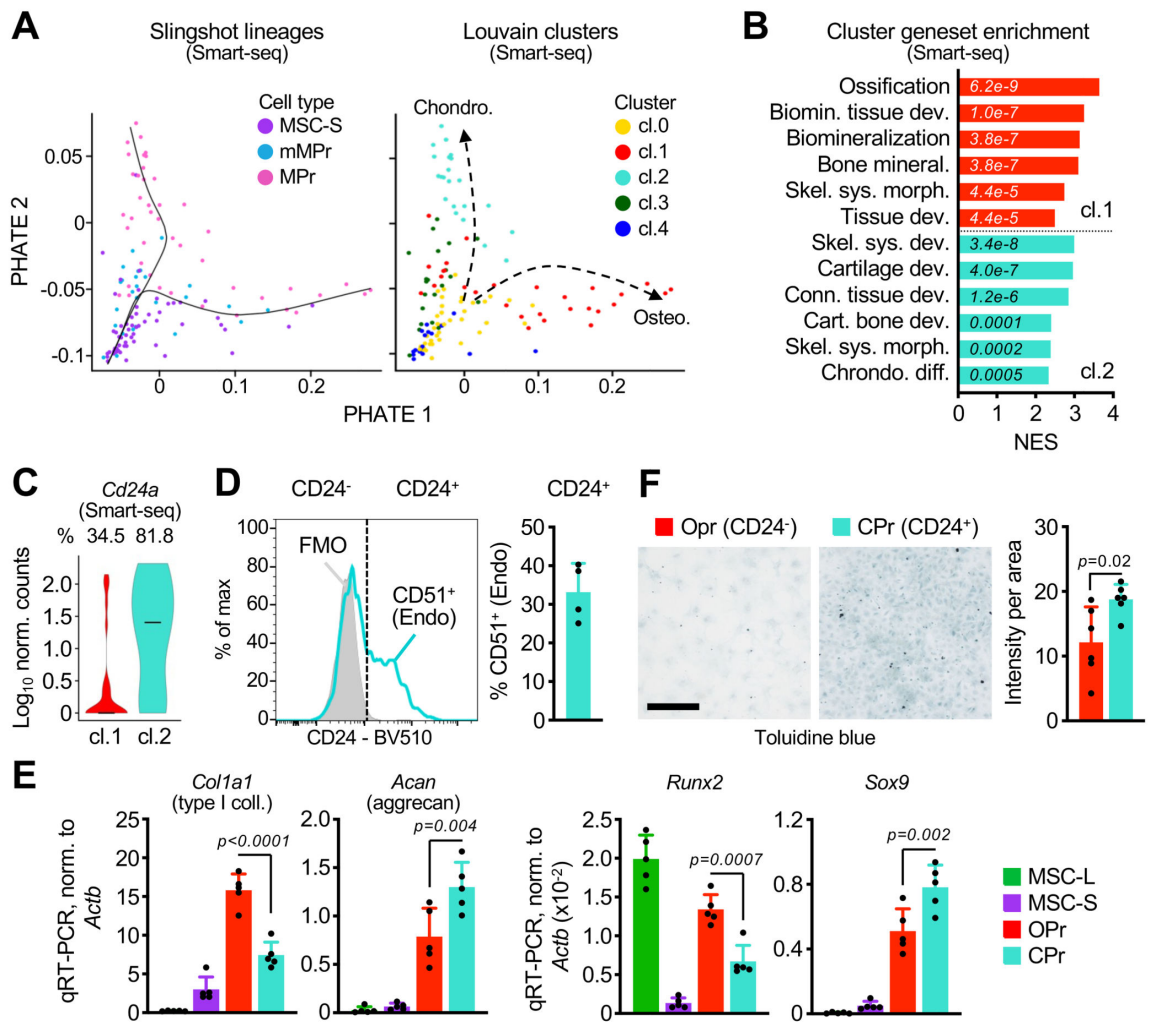
Distinct mesenchymal progenitors generate bone and cartilage cells. **A-C**, Assessment of osteoblastic vs. chondrogenic differentiation from Endo mesenchymal populations: (A) diffusion map created with PHATE from Smart-seq scRNA-seq datasets showing lineage pathways inferred by Slingshot (left) and Louvain clustering (right); (B) GSEA of selectively enriched GO biological pathways (NES: normalized enrichment score, P.adj.: adjusted *P values*); and (C) violin plot showing expression of *Cd24a* and percentage of cells in which expression was detectable in cluster 1 (cl.1) and cluster 2 (cl.2) cells. Lines show median values. **D**, Representative flow cytometry plots (left) and quantification (right) of the proportion of cells expressing CD24 in the CD51^+^ Endo fraction. FMO: fluorescence minus one. **E**, Expression of indicated genes measured by qRT-PCR in flow cytometry-isolated stroma populations. Results are normalized to expression of *Actb*. **F**, Representative images (left) and quantification (right) of chondrocyte differentiation from Endo CD51^+^/CD24^-^ OPr and CD51^+^/CD24^+^ CPr assessed by toluidine blue staining of colonies after 21 days. Results are gray scale intensity values per area. Data are means ± S.D. with points showing values for individual mice (D,E) or MSC colonies (F). *P. values*, derived from permutation tests (B), Student’s t tests (F), or one-way ANOVA with Tukey *post-hoc* test (E).

Projection of our Smart-seq populations onto the mesenchymal atlas also revealed that individual (m)MPr cells were distributed across clusters containing OPr and CPr (Figure 2C). To understand how cells deriving from MSC-Ss differentiate into these two lineages, we produced a diffusion map of MSC-S/mMPr/MPr cells and identified two distinct lineage trajectories using unbiased Slingshot analysis (Figure 3A). Comparison of gene expression revealed a branch significantly enriched for bone formation (cl.1) and another for cartilage development (cl.2) (Figure 3B). Gene expression of cells in cl.1 was highly correlated with the committed osteoblast markers Osx and osteocalcin (Ocn)^49,50^, whereas those in cl.2 were most correlated with primary chondrocytes^51^ (Supplemental Figure 14A). We then searched among DEGs for surface markers that could distinguish both lineages and found expression of the mature chondrocyte marker *Cd24a*^52^ in a higher proportion of cartilage-biased cl.2 progenitors (Figure 3C; Supplemental Figure 14B). Using FC, we confirmed expression of CD24 in a subset of (m)MPr cells, with CD24^+^/CD51^+^ cells having significantly greater expression of the cartilage-specific protein aggrecan (*Acan*) and chondroblast lineage TF *Sox9*, and thus representing CPrs (Figure 3D,E; Supplemental Figure 14C). Conversely, CD24^-^/CD51^+^ (m)MPr had higher expression of bone-associated type 1 collagen (*Col1a1*) and osteoblast TF *Runx2*, thus representing OPrs. Unlike chondrocyte-biased mesenchymal cells in neonatal mice, CD24^+^ CPrs did not have greater expression of CD200 than bone-biased CD24^-^ OPrs, nor did they all express Thy1^[53]^ (Supplemental Figure 14D). Finally, we confirmed that CD24^+^ CPrs showed greater differentiation in conditions promoting chondrocyte maturation than CD24^-^ OPrs, as indicated by toluidine blue staining for proteoglycans (Figure 3F). Collectively, these data confirm the ability of Endo MSC-Ss to differentiate into either osteoblasts or chondroblasts, with surface expression of CD24 enriching for cartilage-biased progenitors in adult mice.

### Inflammation selectively affects MSC-Ls

**Figure 4:**
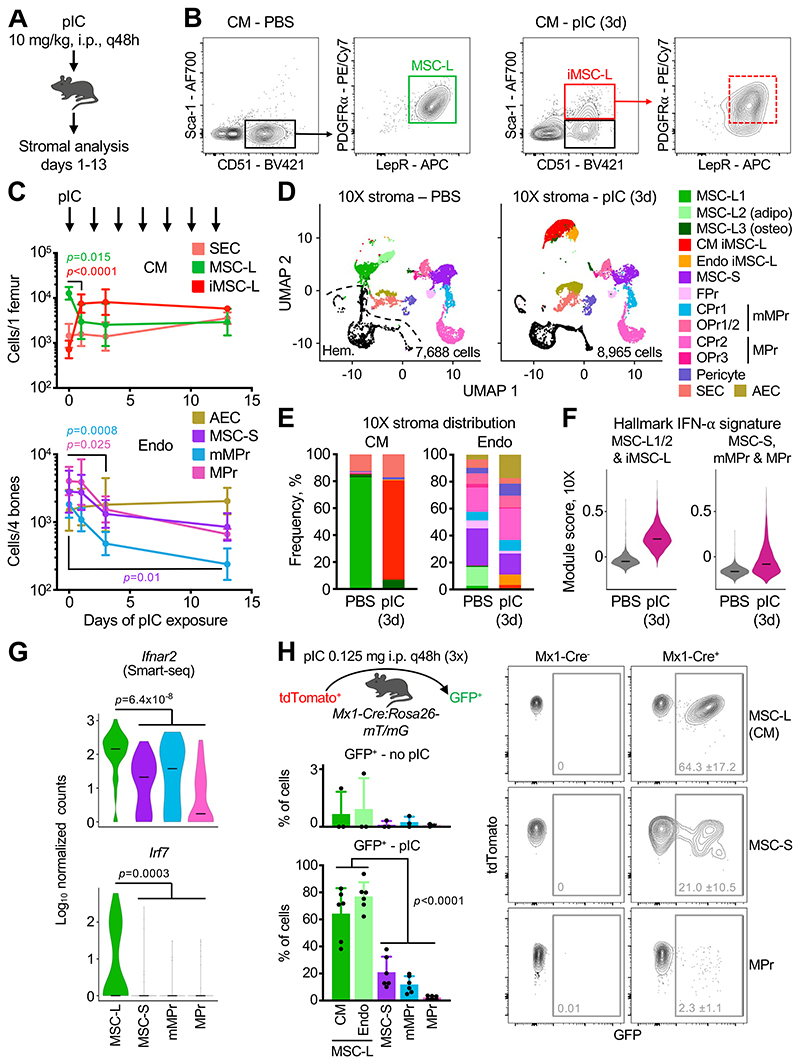
Inflammatory challenge remodels distinct niche compartments. **A-C**, Niche remodeling following interferon-mediated inflammatory challenge: (A) inflammation model produced by repeated injection of polyinosinic:polycytidylic acid (pIC) every second day (q48h) for up to 13 days; (B) representative flow cytometry plots showing increased expression of Sca-1 in inflammatory MSC-Ls (iMSC-L) in the CM of 3 day-pIC-injected mice; and (C) quantification of CM and Endo populations at indicated times following repeated pIC injections. Data are means ± S.D. with n = 3-5 mice per time point. **D-F**, 10X scRNA-seq profiling of CM and Endo fractions isolated from control (n = 1, 2 male mice) and 3 day-pIC-injected (n = 2, 2 male mice each) mice: (D) UMAP of merged datasets with number of analyzed cells and hematopoietic contaminants (Hem.) shown in black; (E) frequency of identified CM and Endo cell types; and (F) gene score for Hallmark interferon (IFN)-α geneset in indicated cell types. **G**, Expression of indicated interferon genes in Smart-seq mesenchymal populations. Data are violin plots of Log10 normalized (norm.) Smart-seq counts with median lines. **H**, GFP conversion upon pIC treatment in *Mx1-Cre:Rosa26-mT/mG* lineage tracing mice with schematic of experimental design (top left), representative flow cytometry plots (right), and quantification (bottom left) of the proportion of cells converting from tdTomato to GFP in the indicated stromal population, with or without pIC treatment. Data are means ± S.D. with points showing values for individual mice. *P. values*, derived from two-way ANOVA with Sidak’s post hoc test (C), one-way ANOVA with Tukey’s *post hoc* test (H), or Wilcoxon rank sum test (G).

We next asked how specific stromal cell populations were affected by inflammation. We treated mice with the toll-like receptor 3 ligand pIC every second day for up to 13 days (Figure 4A) to induce type I interferon (IFN)-dependent inflammation and HSPC activation *in vivo*^54,55^. Remarkably, pIC caused striking upregulation of Sca-1 on MSC-Ls to produce inflammatory MSC-Ls (iMSC-L) that have been observed during aging^4^, and a decline in all Endo mesenchymal populations (Figures 4B,C; Supplemental Figures 15A,B). Inflammatory MSC-Ls were detectable within 3 days of pIC treatment (Figure 4C), and scRNA-seq analyses of niche compartments revealed clustering of iMSC-Ls away from steady state MSC-Ls, demonstrating not just altered expression of *Ly6a*/Sca-1 but a dramatic divergence in the transcriptomic landscape of the entire MSC-L compartment (Figures 4D,E; Supplemental Figure 15C). Given this global change in transcriptional state, the absence of detectable iMSC-L at steady state (Figures 4D,E), and the stable overall number of MSC-Ls in CM (Supplemental Figure 15B), these observations are more consistent with a change in MSC-L cell state, rather than expansion of a pre-existing subpopulation. The preferential response of MSC-Ls to pIC was also associated with greater upregulation of an IFN-α-regulated gene signature in CM/Endo LepR^+^ vs. Endo LepR^-^ mesenchymal cells (Figure 4F). Consequently, we proposed that MSC-Ls might be particularly sensitive to type I IFNs due to their greater resting expression of key components of IFN signal transduction pathway (Figure 4G). To test this notion, we crossed mice carrying the IFN responsive *Mx1-Cre* promoter^56^ with a *Rosa26-mT/mG* reporter^57^ in which Cre recombinase converts tdTomato expression to GFP. No mesenchymal cells expressed GFP at steady state, but, upon pIC exposure, we observed greater GFP expression in both CM and Endo MSC-Ls than in any MSC-S-derived populations, confirming their heightened IFN sensitivity (Figure 4H). Collectively, these data demonstrate that inflammation and IFN signaling specifically affect MSC-Ls.

### iMSC-Ls have reduced osteolineage niche contributions

**Figure 5:**
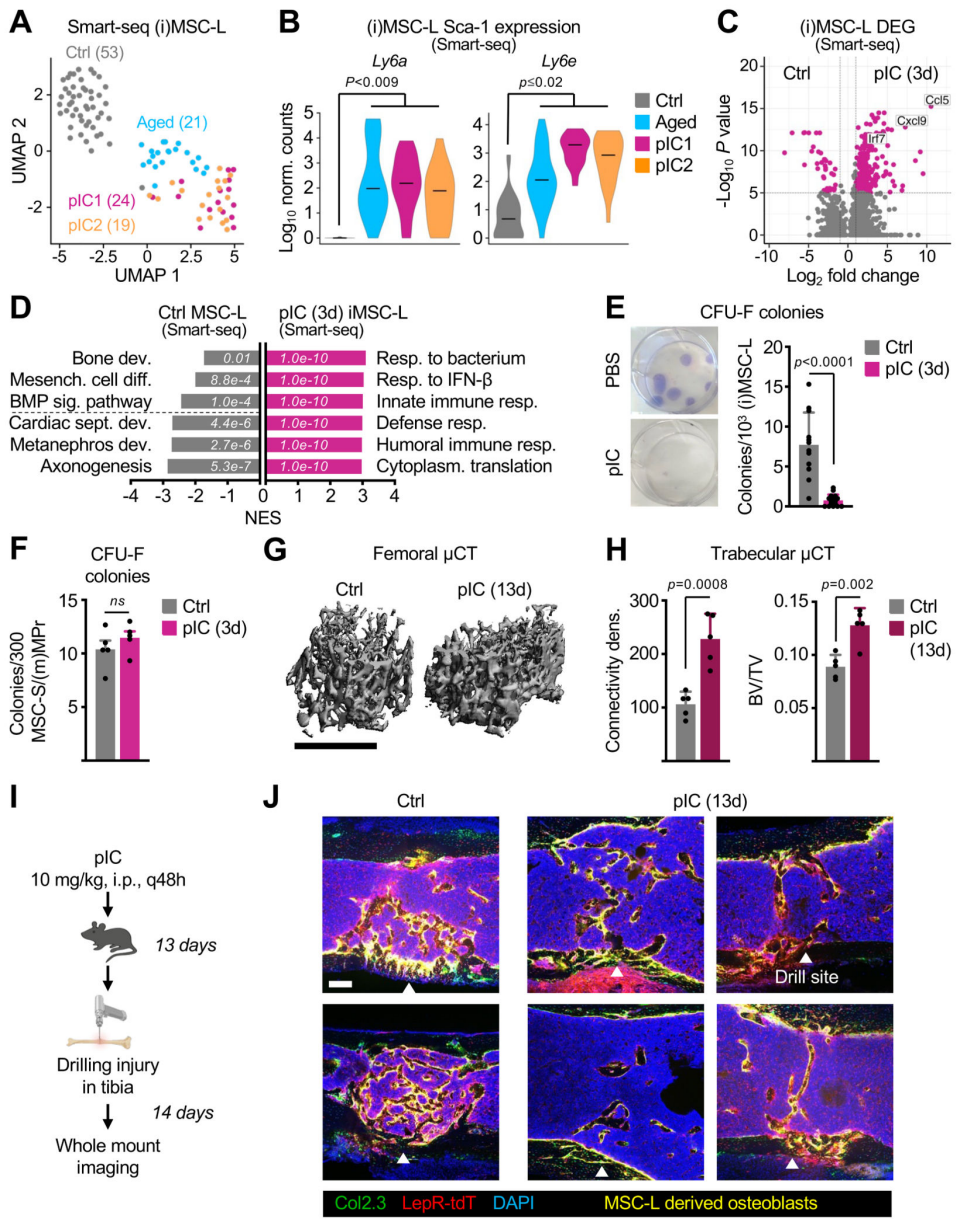
Reduced contributions to stromal niche integrity from iMSC-Ls. **A-D**, Smart-seq scRNA-seq profiling of MSC-Ls isolated from CM of young untreated control mice (Ctrl, n = 3 biological replicates, 2-3 male mice pooled in each replicate) and iMSC-Ls isolated from CM of 24-month-old mice (Old, n = 1 male mouse) and 3 day-pIC-injected young mice (pIC1/2, n = 2 biological replicates, 1 male & 1 female mouse pooled in each replicate): (A) UMAP of merged datasets with number of individual (i)MSC-L cell analyzed; (B) expression of genes encoding Sca-1 constituents (data are violin plots of Log10 normalized (norm.) Smart-seq counts with median lines); (C) volcano plot showing differentially expressed genes (colored points are genes with log_2_ fold change >|1| and adjusted p value <10^-5^); and (D) GSEA of selectively enriched GO biological pathways (NES: normalized enrichment score, P.adj.: adjusted *P values*) in Smart-seq (i)MSC-L dataset. **E**, Representative images (left) and quantification (right) of CFU-F obtained from CM (i)MSC-Ls isolated from 3 days (3d) PBS (Ctrl) or pIC-treated mice (1,000 cells/35-mm well cultured for 8 days in 5% O_2_/iROCK conditions). **F**, Quantification of CFU-F obtained from Endo MSC-S/(m)MPr isolated from 3d Ctrl or pIC-treated mice (300 cells/35-mm well cultured for 11 days). **G-H**, Representative micro-computed tomography images from (G) central femurs and (H) quantification of trabecular bone connectivity density and bone volume/total volume (BV/TV) in mice injected with PBS (Ctrl) or pIC for 13 days (13d). **I**, Effect of 13d pIC treatment on MSC-L response to bone drilling injury with experimental scheme (left) and representative confocal image of tibia whole mount preparation (scale bar: 100 μm) showing bone repair by MSC-L derived osteoblasts (yellow) at the drill site (white arrowheads). Data are means ± S.D. with points showing values for individual mice. *P. values*, derived from Wilcoxon rank sum test (B), permutation tests (D), or Student’s t test (E,F,H).

To understand whether iMSC-Ls could sustain their normal stromal functions, we isolated CM iMSC-Ls from 3 day-pIC-exposed mice for Smart-seq scRNA-seq analyses and functional studies. We first merged these data with Smart-seq analyses of control (Ctrl) CM MSC-Ls from untreated mice and iMSC-Ls isolated from 24-month-old aged mice^4^ (Figure 5A; Supplemental Figure 15D), confirming higher *Ly6a* and *Ly6e* (Sca-1) expression in both pIC-exposed and aged iMSC-Ls (Figure 5B). In addition to IFN signaling and innate immune response pathway upregulation, we also found downregulation of pathways related to mesenchymal differentiation, including bone formation, in pIC-exposed iMSC-Ls (Figures 5C,D). Accordingly, pIC-exposed iMSC-Ls isolated from either CM or Endo fractions could no longer form CFU-F colonies (Figure 5E; Supplemental Figure 15E), which is a prerequisite to assay bone forming capacity, whereas MSC-S-derived Endo cells had no impairment (Figure 5F). However, the trabecular bone mass of mice exposed to pIC over 13 days was unexpectedly increased instead of decreased (Figures 5G,H), which is probably attributable to the parallel role of IFN signaling in inhibiting osteoclast formation^58^. Since MSC-L-derived osteoblasts are involved in fracture repair in adult mice^39^, we asked whether previous pIC exposure would affect MSC-L regenerative responses to cortical injury. We therefore injected pIC or PBS for 13 days in mice bearing a *LepR-Cre* lineage tracing tdTomato reporter^59,60^, created a drill hole in the tibia, and then performed whole mount imaging after 2 weeks^61^. Strikingly, we found that exposure to pIC decreased the number of MSC-L-derived osteoblasts in the fracture site (Figure 5I). Collectively, these results confirm that iMSC-L make diminished contributions to osteolineage cells and stromal niche integrity during inflammation.

### iMSC-L chemokine production regulates monocyte dynamics in the BM niche

**Figure 6:**
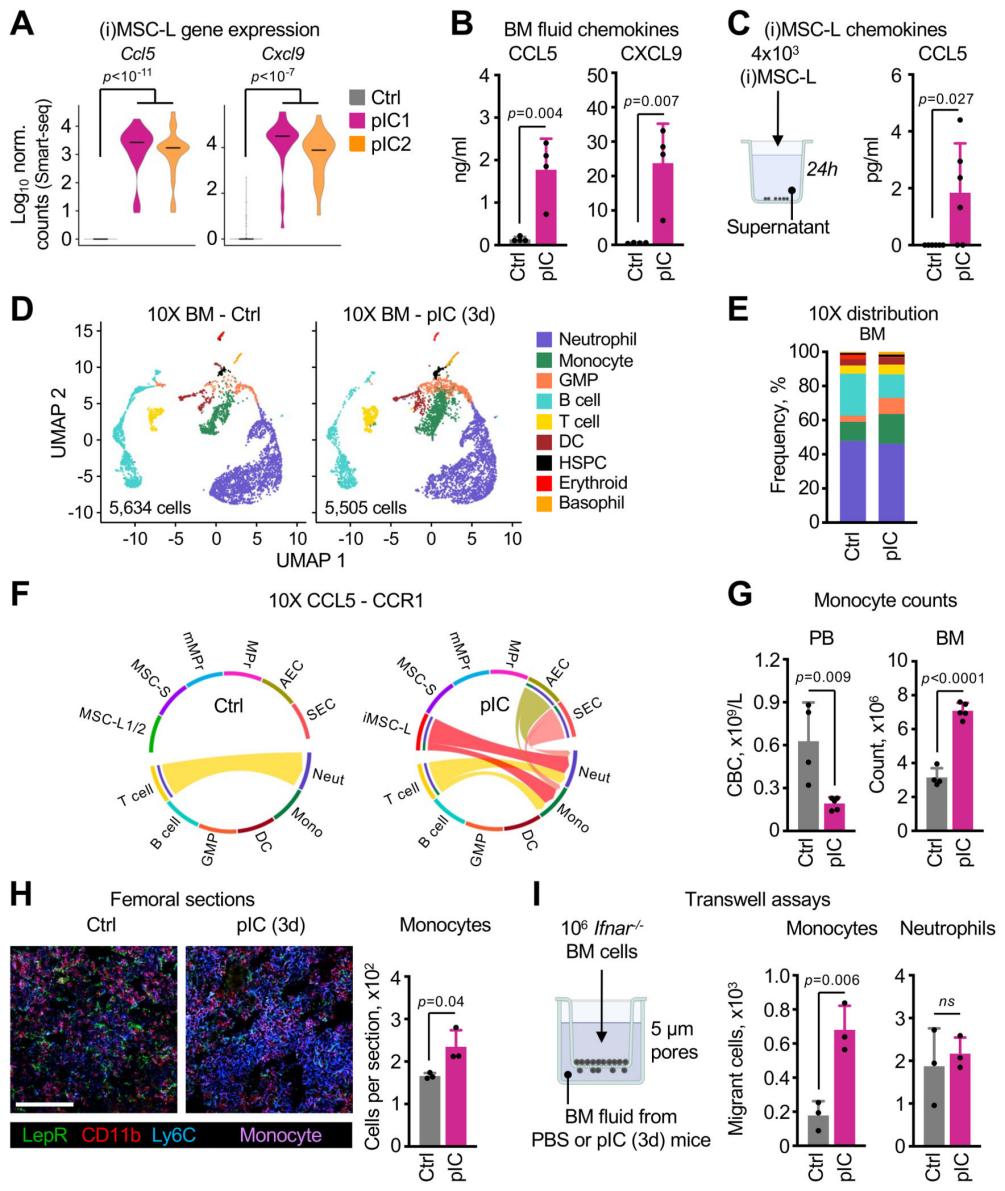
iMSC-Ls modulate local monocytes by chemokine production. **A-C**, Effect of 3 days (3d) pIC treatment on MSC-L chemokine production: (A) expression of *Ccl5* and *Cxcl9* genes in Smart-seq (i)MSC-L dataset (data are violin plots of Log10 normalized (norm.) Smart-seq counts with median lines); (B) production of CCL5 and CXCL9 in BM fluids of 3d PBS (Ctrl) or pIC-treated mice; and (C) measurement of CCL5 in supernatant from 24 hours (24h) cultured (i)MSC-Ls with experimental scheme on the left. **D-E**, 10X scRNA-seq profiling of BM cells isolated from control (n = 1, 2 female mice) and 3d pIC-treated (n = 1, 1 male & 1 female mouse) mice: (D) UMAP of merged datasets with number of analyzed cells; and (E) frequency of identified BM cell types. DC: dendritic cells. **F**, Chord plots showing predicted senders and receivers for CCL5-CCR1 interactions between indicated mature BM and stromal cell types. **G**, Monocyte counts in peripheral blood (PB) and BM of 3d Ctrl or pIC-treated mice. **H**, Representative confocal images of BM sections (left) and quantification (right) of monocytes (scale bar: 50 μm). **I**, Changes in monocyte migration with scheme of the transwell assays (left) using 10^6^ c-Kit-depleted *Ifnar*^*-/-*^ BM cells in the upper chamber and BM fluid from 3d Ctrl or pIC-treated mice in the bottom chamber, and quantification (right) of the number of monocytes and neutrophils migrating into lower chamber after 2 hours (2h). Data are means ± S.D. with points showing values for individual mice. *P. values*, derived from Wilcoxon rank sum test (A), or Student’s t test (B,C,G,H,I).

Among the genes significantly upregulated in pIC-exposed CM iMSC-Ls were the chemokines CCL5 (RANTES) and CXCL9 (MIG) (Figure 6A). We confirmed increased concentrations of both chemokines in BM fluids of 3 day-pIC-exposed mice (Figure 6B), and of CCL5 in the culture supernatant of isolated CM iMSC-Ls (Figure 6C). To understand the impact of MSC-L-derived chemokines on hematopoietic response to pIC, we used CellChat^62^ to explore interactions between stromal and mature BM cells isolated from control and 3 day-pIC-exposed mice (Figures 6D,E). This indicated that iMSC-Ls replaced hematopoietic cells as the predominant producers of CCL chemokines, and predicted that neutrophils and monocytes would be the principal receivers of these signals (Figure 6F; Supplemental Figure 16A). Interestingly, we found marked redistribution of mature CD11b^+^/Ly6C^hi^ monocytes upon pIC treatment, with relative depletion in peripheral blood (PB) and expansion in BM (Figure 6G; Supplemental Figure 16B), which we confirmed *in situ* by immunofluorescent imaging of BM sections (Figure 6H). To determine whether local chemokine production by iMSC-Ls was responsible for selective retention of monocytes in the marrow, we extracted BM fluid from control and 3 day-pIC-exposed mice and evaluated its chemoattractant activity in transwell assays (Figure 6I). Importantly, we used BM cells lacking the type I IFN receptor (*Ifnar*^*-/-*^)^63^ to remove any confounding effect of remaining IFNs. We found that BM fluid from pIC-injected mice specifically attracted monocytes, but not neutrophils, across the transwell membrane to a greater extent than control fluid (Figure 6I), confirming a role for altered MSC-L activity in abnormal monocyte dynamics. To explore the relevance of this finding, we compared gene expression in monocytes from control and 3 day-pIC-injected mice, finding that pIC exposure increased expression of activation markers like cathepsin G and MHC class I (Supplemental Figure 16C), which we confirmed by FC (Supplemental Figure 16D). Additionally, we found that monocytes from pIC-injected mice had significantly greater reactive oxygen species (ROS) content (Supplemental Figure 16E), which was previously associated with HSC activation and enhanced myelopoiesis^64,65^. Together, these results show that pIC-induced production of chemokines by iMSC-Ls attracts and/or retains tissue-toxic monocytes in the BM niche, which might also contribute to IFN-mediated HSPC activation and/or functional impairment.

### The MSC-L inflammatory program is dependent on type I interferon signaling

**Figure 7:**
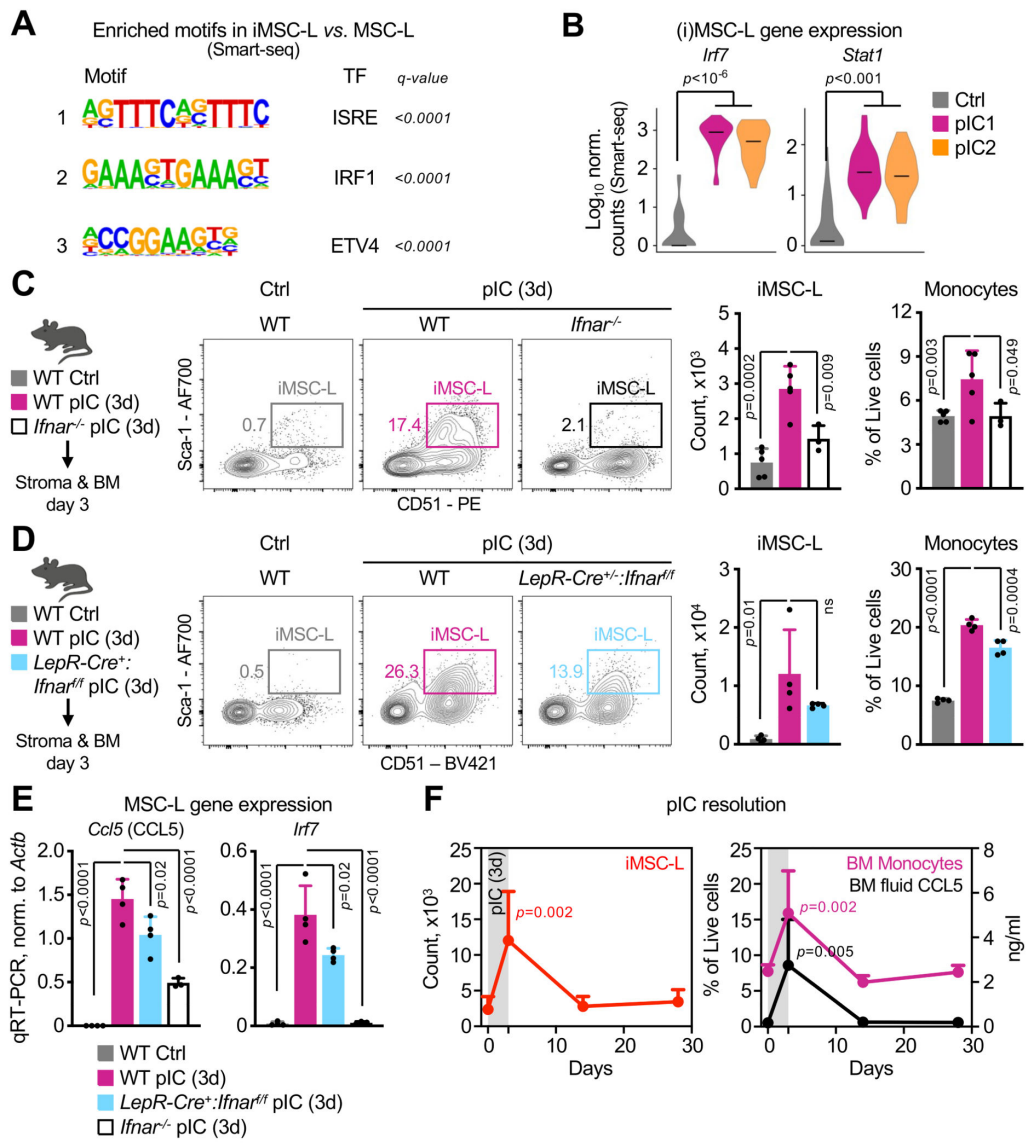
iMSC-L emergence and functions are dependent on type I interferons. **A**, Top 3 HOMER identified transcription factor (TF) motifs enriched in genes differentially expressed in iMSC-Ls. **B**, Expression of *Irf7* and *Stat1* genes in Smart-seq (i)MSC-L dataset. Data are violin plots of Log10 normalized (norm.) Smart-seq counts with median lines. **C**, Mouse lines (left), representative flow cytometry plots (middle), and quantification (right) of iMSC-Ls and monocytes in 3 days (3d) PBS (Ctrl) or pIC-treated wild type (WT) or *Ifnar*^*-/-*^ mice. **D**, Mouse lines (left), representative flow cytometry plots (middle) and quantification (right) of iMSC-Ls and monocytes in 3 days (3d) PBS (Ctrl) or pIC-treated WT or MSC-L-specific *Ifnar*-deleted (*Lepr-Cre*^*+*^:*Ifnar*^*f/f*^ or *Ifnar*^*ΔMSC-L*^) mice. **E**, Ccl5 and Irf7 expression measured by qRT-PCR in the indicated flow cytometry-isolated MSC-Ls. Results are normalized to expression of *Actb*. **F**, Kinetics of iMSC-L resolution with line plots showing evolution in the numbers of iMSC-Ls (left graph), and frequency of BM monocytes as well as CCL5 BM fluid concentration (right graph) following an initial 3d pIC treatment (n = 4-5 mice per time point). Data are means ± S.D. with points showing values for individual mice. *P. values*, derived from Wilcoxon rank sum test (B), or one-way ANOVA with Tukey’s *post-hoc* test (C,D,E,F).

To understand how MSC-Ls are reprogrammed by pIC, we investigated the TF motifs enriched among upregulated genes in iMSC-Ls using HOMER. We found striking enrichment for interferon-stimulated response element (ISRE) motifs, as well as motifs for interferon-response factors (IRF) (Figure 7A), with the gene encoding IRF7 being particularly upregulated in pIC-exposed iMSC-Ls (Figure 7B). Accordingly, we did not observe Sca-1 upregulation, iMSC-L emergence, or monocyte accumulation upon injection of pIC in *Ifnar*^*-/-*^ mice (Figure 7C). To understand the contribution of IFN signaling only in MSC-L, we created conditional knockouts by crossing a *LepR-Cre* strain with *Ifnar*^*f/f*^ mice^63^ (Figure 7D), in which MSC-L *Ifnar* expression was decreased by ~50% (Supplemental Figure 16F). Upon pIC injection, we observed a partial but significant reduction in monocyte accumulation in the BM (Figure 7D), illustrating the direct contribution of MSC-L in this process, as well as decreased *Ccl5* and *Irf7* expression from *Ifnar*-deficient MSC-Ls (Figure 7E). Importantly, injection of pIC in *Ifnar*^*-/-*^ mice did not increase *Irf7* expression in MSC-L, confirming that the type I IFN pathway was not activated (Figure 7E). However, *Ifnar*^*-/-*^ MSC-L displayed some *Ccl5* expression in response to pIC, suggesting that secondary factors besides type I IFNs could drive some elements of the iMSC-L gene expression program (Figure 7E). Moreover, upon pIC injection in *LepR-Cre:Ifnar*^*f/f*^ mice, there were no differences in monocyte activation shown by MHC expression or ROS content (Supplemental Figure 16G), which likely result from direct effects of IFNs on monocytes. Lastly, we found that the stromal response to pIC dissipated upon withdrawal, with iMSC-Ls no longer observed in the CM, monocytes not expanded, and CCL5 levels returning to baseline two weeks after the last injection (Figure 7F). Collectively, our data suggest that the enhanced IFN sensitivity of MSC-Ls poises them to act as first responders to IFN challenges in the BM niche, transducing these pathogenic signals through production of chemokines to affect the trafficking of inflammatory monocytes in and out of the marrow.

## Discussion

Here, we present a unified scheme for stromal cell identification and isolation, which we cross-reference to previous datasets to show that our annotation captures most major cell types in the BM niche and aligns well with previous imaging and functional studies. Moreover, our analysis posits the existence of two distinct but converging sources of bone-forming cells from either CM/Endo MSC-Ls or Endo MSC-Ss, which overlaps with recent findings on the roles of different skeletal stem cells in fracture repair^66^. We also find that at steady state, only MSC-Ss and their derivatives are predicted to have fibroblastic and chondroblastic potential, whereas MSC-Ls alone are predicted to generate adipocytes *in vivo*, even though MSC-Ss and MSC-Ls can both be induced to form CFU-F *ex vivo*. Our work further implies that different mesenchymal lineage fates are largely partitioned by spatial location in the BM niche in healthy adult mice, though others have shown that lineage restriction may be negotiable under stress conditions, such as when CM MSC-Ls produce chondroblasts during fracture repair^39^, or when Endo MSC-Ss produce occasional adipocytes upon transplantation^40^. Finally, comparison of transcriptomic profiles between murine and human stromal cells suggests strong conservation of these two distinct MSC populations, including their spatial organization and lineage potentials^29^, which highlights the utility of mouse models for understanding BM niche responses to inflammation.

We find that MSC-Ls, whether in CM or Endo, are exquisitely sensitive to IFN challenges, though we cannot exclude the possibility that some elements of the iMSC-L phenotype are driven by secondary factors. Prior studies reported that MSC-Ls respond to pIC and viral infections^3,26^, and we now show that iMSC-L forego mesenchymal differentiation in favor of local inflammatory chemokine production, which affects monocyte distribution. While CCL2 production by MSC-Ls was already implicated in monocyte egress from BM^67^, we show how the chemokine landscape can be altered by type I IFNs, leading to production of CCL5 and CXCL9 that retain inflammatory monocytes in the BM. This finding complements our recent work in aged mice^4^, where prolonged exposure to IFNs in the aging niche could be implicated in the functional impairment of aged MSC-Ls to the presumed detriment of nearby HSPCs^68^. In practical terms, we confirm that MSC-Ls are the primary stromal cell population affected by *Mx1*-Cre, which is an important confounding factor for use of this model system when studying HSC functions^69,70^.

Collectively, our work reconciles numerous studies of the BM niche into a unified scheme that incorporates spatial compartmentalization and provides a method for investigating the effect of inflammation on both hematopoietic and stromal compartments. Further studies can now investigate how stromal niche compartmentalization is established and maintained, whether different bones or different regions of the same bone show different stromal organization, and how stromal responses might be manipulated therapeutically to modulate hematopoietic responses in inflammation and cancer.

## Supplementary Material

Supporting Information

Supporting Information 2

Supporting Information 3

## Data Availability

Datasets that support the findings of this study have been deposited in the Gene Expression Omnibus (GSE276784), reviewer token **sfezckcqbpmzdiz**. Source data for all the figures are provided with the paper. All other data are available from the corresponding author upon reasonable request.

## Key Points

- Murine bone marrow is compartmentalized into central marrow and endosteal niches, enriched for distinct hematopoietic and stromal cells.
- Inflammation specifically targets leptin receptor-expressing mesenchymal stromal cells, causing them to secrete chemokines affecting monocyte dynamics.
