# Supplementary material for: Inflammation perturbs hematopoiesis by remodeling specific compartments of the bone marrow niche
Source: Blood. Author manuscript; Available in PMC 2025 Dec 10. (PMC7618457; doi:10.1182/blood.2025029513)
Supplement: Supporting Information [file EMS211406-supplement-Supporting_Information.pdf]

Supplemental Methods

Mice

All animal experiments were conducted at the University of California San Francisco (UCSF) or Columbia University Irving Medical Center (CUIMC) in accordance with Institutional Animal Care and Use Committee protocols approved at each institution, and in compliance with all relevant ethical regulations. Young wild type (WT) C57BL/6-CD45.2 mice were purchased from Jackson Laboratory and bred in house. Aged WT C57BL/6-CD45.2 mice were obtained from the National Institute on Aging (NIA) when they were 18 months old and used for experiments when they were 24 months old. BALB/CJ, *Ifnar1*$^{-/-}$ (63), *Ifnar1*$^{f/f}$ (63), *Mx1*-Cre[56], and mTmG reporter mice[57] were purchased from the Jackson Laboratory. For mouse strains expressing fluorescent reporters, *Vegfr3*-YFP[30] and *CX40*-GFP[34] mice were shared from the laboratory of Dr. Jason Butler at the University of Florida. *Nestin*-GFP[44] mice were shared from the laboratory of Dr. Maria Maryanovich at Albert Einstein College of Medicine. *Col2.3*-GFP, *Lepr-Cre*:Lox-STOP-Lox-tdTomato[59,60], *Lepr*-Cre:ROSA26-YFP[59,60] and *Prx1*-Cre:ROSA26-tdTomato[43] mice were shared from the laboratory of Dr. Lei Ding at CUIMC. *Osterix*-GFP[48] mice were shared from the laboratory of Dr. Stavroula Kousteni at CUIMC. *Hes1*-GFP mice were shared from the laboratory of Dr. Iannis Aifantis at New York University. Mice were 8 to 12 weeks of age when used for experiments. No specific randomization or blinding protocol was used with respect to the identity of experimental animals, and both male and female animals were used in all experiments.

In vivo assays

For polyinosinic:polycytidylic acid (pIC, Cytiva) treatment, pIC was dissolved in sterile PBS at 1.25 mg/ml and then mice were injected intraperitoneally with 10 mg/kg pIC every 48h, with BM and stroma analyses performed on days 1-13 after starting pIC treatment. For dragon green bead (DGB, Bangs

Laboratories, FSDG001) analyses, mice were injected retro-orbitally with 2.5 μl/g DGB solution 10 minutes before euthanasia and then perfused with 20 ml PBS by cardiac puncture before collecting the bones for analysis.

Combined stromal and hematopoietic cells isolation

Mice were euthanized in a rising concentration of carbon dioxide, followed by cervical dislocation. For each mouse, both femurs, tibiae, hemipelves, and humeri were dissected and thoroughly cleaned using KimWipes (Kimtech) and were used for combined isolation of stromal and hematopoietic cells. To isolate central marrow (CM) stromal cells, the proximal and distal epiphyses were removed from 1 femur. Intact marrow plugs were flushed with Hank's balanced saline solution (HBSS) without calcium or magnesium into 5 ml polypropylene tubes by inserting a 3 ml syringe with 22G needle into the distal end of the femur. The plugs were digested with 1 ml of a solution of 3 mg/ml type I collagenase (Worthington) dissolved in HBSS for 10 min at 37 °C with 110 rpm shaking. The tube was then vortexed briefly and the supernatant was removed into a new tube through a 100 μm mesh, taking care not to disturb the marrow plug. A further 1 ml of 3 mg/ml type I collagenase solution was then added to the marrow plug and the digestion repeated. After the second incubation, the plug was dissociated by pipetting up and down with a P1000 pipette before passing the cell suspension through a 100 μm mesh into the tube containing the previous digestion supernatant. To isolate endosteal (Endo) stromal cells and hematopoietic cells, the flushed femur was combined with the other bones, gently crushed up to 10 times using a mortar and pestle, and thoroughly washed with 10 ml HBSS until all the non-adherent BM cells were removed and collected in a separate 15 ml polypropylene tube. The bone chips were then digested in a 15 ml polypropylene tube with 3 ml of 3 mg/ml type I collagenase solution for 1 hour at 37 °C with 110 rpm shaking. After digestion, the tube was vortexed briefly and the cell suspension was filtered through a 100 μm mesh into a new tube. The

bone chips were then washed with HBSS, and the washing was collected into the same tube as the digestate. For cell suspensions acquired for both stromal compartments, red blood cells were removed by adding 1 ml ACK lysis buffer (150 mM NH_4Cl and 10 mM $KHCO_3$) and incubated on ice for 3 minutes before washing with HBSS containing 4% fetal bovine serum (HI FBS, Gibco). During the optimization of the protocol outlined above, the digestion with 3 mg/mL type I collagenase was performed in parallel to and compared with 3 mg/mL type I collagenase + 4 mg/mL type II Dispase (Roche), 250 μg/mL Liberase DL (Roche) + 200 U/mL DNAse I (Sigma), and mechanical dissociation with P1000 pipette. The BM cells collected from crushed bones were resuspended in HBSS with 2% FBS and RBCs were removed by lysis with ACK buffer. The BM cell suspension was further purified on a density gradient (Histopaque 1119, Sigma-Aldrich), by layering 2 ml of the cell suspension under 2 ml of Histopaque solution. For profiling of CM plug and Endo marrow, only femurs were used and were flushed once with a 3 ml syringe and 21G needle to collect BM plugs, while the flushed bones were crushed to collect the remaining Endo-associated BM cells. No ACK RBC lysis was performed for these analyses. Both stromal and hematopoietic cells were finally counted using a Vicell automated cell counter (Beckman Coulter).

Flow cytometry of stromal cells

For stromal cell profiling, Endo and CM cell preparations were stained with CD45-APC/Cy7 (BD, 557659; 1:400), Ter119-PE/Cy5 (Invitrogen, 15-5921-83; 1:400), Sca-1-AF700 (eBioscience, 56-5981-82; 1:800), CD31-PE (BD, 553373; 1:200), CD105-BV786 (BD, 564746; 1:200), CD51-BV421 (BD, 740062; 1:100), LepR-biotin (R&D, BAF497; 1:100), and PDGFRα-PE/Cy7 (Invitrogen, 25-1401-82; 1:100) for 30 minutes on ice. Cells were then washed and stained with Streptavidin-APC (BioLegend, 405207;1:400) for 15 minutes on ice. For analysis of CD24 expression, CD24-BV510 (BioLegend, 101831; 1:400) was added to the staining panel. For the analysis of endomucin, endomucin-APC (Invitrogen, 50-5851-80; 1:100) was used and LepR antibody was dropped from the staining panel. For

the analysis of CD34, CD34-FITC (eBioscience, 11-0341-85; 1:25) was added to the staining panel. For the analysis of NG2, cells were incubated with unconjugated monoclonal rabbit anti-mouse NG2 antibody (Millipore Sigma) alongside other conjugated antibodies, then washed and stained with goat anti-rabbit secondary antibody conjugated to AF488 (Invitrogen, 1:400) for 30 minutes. For analysis of chondrocyte markers, Thy1-APC (combination of BioLegend, 166405 and BioLegend 105311, 1:200), 6C3-FITC (BioLegend, 108305, 1:200), and CD200-PE/Dazzle (BioLegend, 123819, 1:200) were added to the panel. Cells were finally washed and resuspended in HBSS with 4% FBS and 1 μg/ml propidium iodide and then analyzed or sorted on purity mode on a BD FACSAria II (UCSF) or FACSAria II SORP (CUIMC). Data collection was performed using FACSDiva (v.9) and analysis was performed using FlowJo (v.9/v.10).

Flow cytometry of hematopoietic cells

For HSPC profiling, BM cells were stained with c-Kit-APC-Cy7 (BioLegend, 105826; 1:800), Sca-1-BV421(BioLegend, 108128; 1:400), CD150-BV650 (BioLegend, 115931; 1:200), CD48-A700 (BioLegend, 103426; 1:400), Flt3-PE (eBioscience,12-1351-82; 1:100), CD34-FITC (eBioscience, 11-0341-85; 1:25) and CD16/32-PE/Cy7 (BioLegend, 101318; 1:800), along with lineage antibodies in PE/Cy5 (Gr1 [eBioscience, 15-5931-82; 1:800], CD11b [Invitrogen, 15-0112-82; 1:800], B220 [Invitrogen, 15-0452-82; 1:800], CD19 [BioLegend, 115510; 1:800], CD5 [BioLegend, 100610; 1:800], CD4 [Invitrogen, 15-0041-82; 1:800], CD8a [Invitrogen, 15-0081-82], Ter119 [Invitrogen, 15-5921-83; 1:400], CD3 [Invitrogen, 15-0031-83; 1:400]). For mature cell profiling, cells were stained with Ly6G-FITC (BioLegend, 127606, 1:400), Ly6C-APC (BioLegend, 128016, 1:400), CD8-AF700 (BD, 557959, 1:400), B220-APC/Cy7 (BioLegend, 103224, 1:800), CD4-BV510 (BioLegend, 100553, 1:400), CD11b-BV786 (BioLegend, 101243, 1:800), CD115-PE (BioLegend, 135506, 1:100), and CD3-PE/Cy7 (BioLegend, 100220, 1:100). For some analyses, cells were also stained with H2-Kb-FITC (MHC class I, BioLegend, 116505, 1:100) or CD16/32-PE/Cy7 (BioLegend, 101318, 1:800). For DHR123 staining, after

surface staining, cells were re-suspended in DHR123 (Invitrogen, D23806) at 1 μM in HBSS and incubated for 30 minutes at 37°C. Cells were finally washed, resuspended in HBSS with 2% FBS and 1 μg/ml propidium iodide, and analyzed on an Agilent Novocyte Quanteon or Penteon (CUIMC). Data analysis was performed using FlowJo (v.9/v.10).

Stromal and hematopoietic cell isolation for 10X scRNA-seq analyses

For sequencing of Endo and CM stromal cell preparations, 30,000-50,000 Ter119^{-}/CD45^{-} cells were sorted into 1.5 ml tubes containing 300 µl of 50% FBS and 50% HBSS. For sequencing of BM cells, 40,000 live (propidium iodide negative) cells were similarly isolated from PBS or pIC-injected mice. For sequencing of defined stromal populations, oligo-hashed antibodies specific for MHC class I and CD45 (BioLegend, TotalSeq B1-10) at a concentration of 0.375 μg of antibody per 10^6 cells were mixed with surface antibodies during stromal cell isolation from separate samples of CM or Endo preparations (1 TotalSeq B antibody per sample type). Cells were then washed three times with HBSS containing 4% FCS and the indicated stromal populations were isolated by flow cytometry and mixed according to the following scheme to produce two separate combined stains, each containing a total of 25-40,000 cells:

Sample type	Oligo hash antibody	Combined stain 1 (cells)	Combined stain 2 (cells)
CM	TotalSeq B1	MSC-L (6899)	SEC (2228)
CM	TotalSeq B2	mMPr (2775)	SEC (2097)
CM	TotalSeq B3	mMPr (2406)	SEC (2529)
CM	TotalSeq B4	MPr (847)	SEC (1041)
CM	TotalSeq B5	MPr (802)	SEC (1353)

CM	TotalSeq B6	MSC-L (7642)	AEC (438)
CM	TotalSeq B7	-	AEC (496)
Endo	TotalSeq B8	MPr (10048)	MSC-S (8212)
Endo	TotalSeq B9	AEC (5046)	MSC-L (2319)
Endo	TotalSeq B10	SEC (3537)	mMPr (6823)

In all cases, following isolation, cells were rested for 1 hour on ice then pelleted at 350 x *g* for 5 minutes at 4°C before the supernatant was removed down to a volume of 40 µl. GEM generation and 3' RNA library preparation was performed according to 10X Genomics protocol CG000315 Rev E, targeting 5000 cell data recovery. RNA libraries were pooled 1:1:1:etc, sequenced on an Illumina NovaSeq 5000, and aligned using Cellranger (v.7.0.1) to mouse genome mm10. For hashing samples, hashing libraries were pooled with RNA libraries at a ratio of 1:4. Library concentrations and fragment sizes were evaluated using Qubit dsDNA HS assay kit (ThermoFisher Scientific) and TapeStation D5000 DNA ScreenTape analysis (Agilent).

Stromal cells and MSC-L isolation from Smart-seq scRNA-seq analyses

For Smart-seq analyses of Endo (AEC, MSC-S, mMPr, MPr) and CM (SEC, MSC-L) stromal cells isolated from young WT mice, we utilized our own published data[4] that was processed using the same analytical pipeline. For Smart-seq of iMSC-L isolated from 3 day-pIC-injected young WT mice and 24-month-old aged WT mice, we utilized the SMART-Seq Single Cell PLUS kit (96 reactions, Takara Bio). Single iMSC-L were sorted into 96 well plates containing 12.5 µl of CDS sorting solution provided in the kit. Preparation and amplification of cDNA and single cell libraries were completed according to the manufacturer's instructions. Single cell libraries that passed quality control (n=43 from pIC-injected mice and n=30 from aged mice) were pooled at a 1:1:1:etc ratio before sequencing on an Illumina NextSeq

500/550 instrument using a version 2 kit (Illumina). Fastq files were generated for each library in Illumina BaseSpace, and quality of data was evaluated with FastQC and MultiQC[71]. After removing samples with poor sequencing quality, 43 cells from pIC-injected mice and 21 cells from aged mice were used for analysis. After trimming adapters with TrimGalore[72], sequencies were pseudoaligned to the murine mm10 genome and counted using Salmon[73].

10X scRNA-seq data analyses - CM and Endo preparations

Count matrices for 10X scRNA-seq were analyzed with Seurat (versions 4 and 5)[74,75] in R (version 4.3.1). For CM and Endo CD45^{-}/Ter119^{-} stromal samples, we observed higher counts of ambient RNA in some samples, so we adjusted the matrices for all stromal samples using the R package SoupX[76] with default parameters. To exclude damaged cells or probable doublets, we filtered cells according to the following criteria after creation of Seurat objects: mitochondrial reads per cell >5.5%, number of reads per cell >65,000, number of unique genes per cell <1000 and >7000. For generation of the reference 10X stroma map, we merged the CM and Endo sequencing results of three independent datasets, normalized the data using the *SCTransform* function, and then integrated these samples together using SCT integration functions (*SelectIntegrationFeatures*, *PrepSCTIntegration*, *FindIntegrationAnchors*, *IntegrateData*). We performed principal component analysis (*RunPCA*, 35 components) before creating UMAP visualization (*RunUMAP*, 17 dimensions) and discovering Louvain clusters (*FindNeighbors*, 30 dimensions, *FindClusters*, resolution 0.5). We removed a single cluster that contained cells overlapping with numerous other clusters in the UMAP visualization, which probably represented a small number of residual cells of poor quality. We mapped Smart-seq dataset of stromal cells and 10X scRNA-seq of purified stromal populations to this 10X stroma reference map using *FindTransferAnchors* and *MapQuery* functions. We annotated clusters and assigned names manually in the scRNA-seq reference dataset by (i) evaluating key marker genes identified using the *FindAllMarkers* function, (ii) mapping our Smart-seq dataset, and (iii)

mapping our purified 10X scRNA-seq samples. We evaluated correlations between clusters in the reference 10X stroma map and Smart-seq datasets by generating pseudobulk RNA profiles for each Smart-seq population (using the *AggregateExpression* function) then using the *clustify* function from the ClustifyR package[77] to calculate spearman correlations for each cluster in the reference 10X stroma map with the top 2,000 highly variable genes. For heatmap visualization, we scaled these correlations between 0 and 1 for each Smart-seq cell type. For analysis of the impact of pIC on stromal cells, we merged the sequencing results from control and 3 day-pIC-exposed mice to generate a complete dataset, before applying the same quality control filters and analytical functions as described above. To evaluate changes in gene expression within clusters upon pIC exposure, we used the *FindMarkers* function (minlog2foldchange 0.25, min.pct 0.25). To evaluate the type I IFN response in stromal cells, we scored cells in the merged dataset using the *AddModuleScore* function, utilizing the Hallmark Interferon Alpha Response v.7.5.1 signature. Geneset enrichment analysis was performed using the GO biological pathways with the *gseGO* function from clusterProfiler[78,79] (OrgDb = org.Mm.eg.db, ont = "BP", minGSSize = 10, maxGSSize = 800, pvalueCutoff = 0.05, pAdjustMethod = "none").

10X scRNA-seq data analyses - BM cells

Count matrices for 10X scRNA-seq were analyzed with Seurat (versions 4 and 5) in R (version 4.3.1). To exclude damaged cells or probable doublets, we filtered cells according to the following criteria after creation of Seurat objects: mitochondrial reads per cell >10%, number of unique genes per cell <200. To evaluate the effect of pIC on mature BM cells, we normalized control and pIC datasets using the *SCTransform* function, then integrated these samples together using SCT integration functions (*SelectIntegrationFeatures*, *PrepSCTIntegration*, *FindIntegrationAnchors*, *IntegrateData*). We scored the cell cycle status of these cells (*CellCycleScoring*) before regressing the effects of the S and G2M scores. We performed principal component analysis (*RunPCA*, 30 components) before creating UMAP

visualization (*RunUMAP*, 30 dimensions) and discovering Louvain clusters (*FindNeighbors*, 30 dimensions, *FindClusters*, resolution 0.5). We annotated clusters using characteristic marker genes found with the *FindAllMarkers* function, and we evaluated the effect of pIC within clusters using the *FindMarkers* function (minlog2foldchange 0.25, min.pct 0.25).

10X scRNA-seq data analyses - Oligo-hashed stromal cells and HSPCs

Count matrices for 10X scRNA-seq were analyzed with Seurat (versions 4 and 5) in R (version 4.3.1). The RNA data for oligo-hashed stromal cells were filtered and analyzed as described in the preceding sections. Hashing data for stromal datasets were normalized using *NormalizeData* then deconvoluted using *MULTIseqDemux* to assign labels for each cell and merged into a single Seurat object.. For oligo-hashed stromal cells, since the frequency of successful hash labels was rather low across the 2 combined stains we generated, we utilized a combination of information to identify CM and Endo MSC-Ls, SECs, and AECs, including: any available hashtag labels, the presence of a cell type in each sample compared to the expected composition, and the presence of characteristic gene markers identified with the *FindMarkers* function. Data from the purified stromal samples were mapped to reference 10X stroma map using *FindTransferAnchors* and *MapQuery* function.

10X scRNA-seq data analyses – CellChat interactome

Cells corresponding to major stromal populations (MSC-L1, MSC-L2, MSC-S, mMPr, MPr, SEC, AEC, iMSC-L) were extracted from the 10X stroma samples from control and pIC exposed mice, as well as mature hematopoietic cells from control mice (neutrophils, monocytes, dendritic cells, granulocyte macrophage progenitors, B cells, and T cells). Raw counts from hematopoietic and stromal cells were then merged into a single Seurat object. Counts were normalized with *SCTransform*, and the normalized matrix was extracted to create a CellChat object[62] using *createCellChat*, which was then processed using standard

functions and default settings, selecting only "Secreted Signaling" for analysis. We used the function *netAnalysis_signalingRole_heatmap* to create heatmaps showing major incoming and outgoing signals for included cell types across significantly enriched signaling families. We then used *mergeCellChat* to create a composite object, followed by *netAnalysis_signalingRole_network* and *netVisual_individual* for visualization of important pathways.

Smart-seq scRNA-seq data analyses

The counts table derived from Mitchell et al. (2023)[4] was imported into Seurat (versions 4 and 5) in R (version 4.3.1), with no additional filtering. Counts were processed using *NormalizeData*, *FindVariableFeatures*, and *ScaleData*. Differences in gene expression between stromal populations were evaluated using *FindMarkers* (minlog2foldchange 0.25, min.pct 0.25). For evaluation of differentiation trajectories among endosteal stromal cells, MPr, mMPr, and MSC-S cells were extracted to generate a diffusion map using the function *phate* in the R package PhateR[80]. Dimension reduction and clustering were repeated on this dataset using the following functions and settings: *RunPCA* (30 dimensions), *RunUMAP* (20 dimensions), *FindNeighbors* (20 dimensions), *FindClusters* (resolution = 1.0). The function *slingshot* from the R package Slingshot[81] was then used to identify trajectories in the PHATE dimension reduction, specifying cluster 4/MSC-S group as the starting node, and these trajectories were visualized using *getLineages* and *getCurves* functions before plotting in base R. Geneset enrichment analysis was performed using the GO biological pathways with the *gseGO* function from clusterProfiler (OrgDb = org.Mm.eg.db, ont = "BP", minGSSize = 10, maxGSSize = 800, pvalueCutoff = 0.05, pAdjustMethod = "none"). For evaluation of the impact of aging and pIC on MSC-Ls, young control MSC-L data were downloaded from Mitchell et al. (2023)[4], reprocessed, and merged with the pIC/aging iMSC-L Smart-seq counts generated in this study. These counts were then imported into Seurat (versions 4 and 5) in R and processed using *NormalizeData*, *FindVariableFeatures,* and *ScaleData*. We performed

principal component analysis (*RunPCA*, 30 components) before creating UMAP visualization (*RunUMAP*, 20 dimensions) and discovering Louvain clusters (*FindNeighbors*, 20 dimensions, *FindClusters*, resolution 1.0). Differentially expressed genes were evaluated using *FindMarkers* (minlog2foldchange 0.25, min.pct 0.25), and volcano plots were generated using the *EnhancedVolcano* function from EnhancedVolcano. Geneset enrichment analysis was performed using the GO biological pathways with the *gseGO* function from clusterProfiler (OrgDb = org.Mm.eg.db, ont = "BP", minGSSize = 10, maxGSSize = 800, pvalueCutoff = 0.05, pAdjustMethod = "none"). Evaluation of transcription factor motifs in differentially expressed genes was performed using HOMER[82], running the *findMotifs.pl* function in the command line with default parameters for mouse promoters.

Analyses of published 10X scRNA-seq datasets

To generate the 10X mesenchymal atlas, data were obtained from three previous studies: Baccin et al., 2020 [GSE122465][17]; Baryawno et al., 2019 [GSE128423][18]; Zhong et al., 2020 [GSE145477][41]. For GSE122465, the counts table and metadata (including principal components) were downloaded directly, with no further filtering. For visualization, the function *RunUMAP* (20 dimensions) was performed. For GSE128423, 6 files corresponding to stromal cells (GSM3674224, GSM3674225, GSM3674226, GSM3674227, GSM3674228, GSM3674229) were downloaded and read into Seurat (versions 4 and 5) in R (version 4.3.1) then merged into a single object. Low quality cells were filtered according to the following criteria: mitochondrial reads per cell >5.5%, number of reads per cell >65,000, number of unique genes per cell <200 and >7000. The merged object was then processed using the following functions: *NormalizeData*, *FindVariableFeatures*, *ScaleData*, *RunPCA* (30 dimensions), *RunUMAP* (20 dimensions), *FindNeighbors* (20 dimensions), *FindClusters* (resolution 0.5). Since the original cell annotation was not available in the submission, the top 50 differentially expressed genes for each cluster were obtained from *Supplementary Data 1* of Baryawno et al. (2019)[18], and the function

SCINA from the R package SCINA[83] was used to predict the identity of every cell, using the same nomenclature shown in *Figure 1* of Baryawno et al. (2019)[18]. For GSE145477, the count tables derived from 1 month old (GSM4318799) and 1.5-month-old (GSM4318800) mice were downloaded and used to create a merged Seurat object. The cells were then filtered and processed as described for GSE128423. For creation of the 10X mesenchymal atlas, clusters corresponding to endothelial cells or hematopoietic cells were manually identified in each component dataset using characteristic marker genes based on the *FindAllMarkers* functions, and these cells were removed. The raw counts for the remaining mesenchymal cells were normalized separately using *SCTransform* then integrated using SCT integration functions (*SelectIntegrationFeatures*, *PrepSCTIntegration*, *FindIntegrationAnchors*, *IntegrateData*). The integrated assay was then processed using *RunPCA* (30 components), *RunUMAP* (20 dimensions), *FindNeighbors* (20 dimensions), and *FindClusters* (resolution 0.5). For trajectory inference, functions from Monocle3 were implemented through SeuratWrappers[84]. The *learn_graph* and *order_cells* functions were run twice using two different starting nodes corresponding to different MSC populations; LepR$^+$ MSCs and Sca-1$^+$ MSCs points were defined using genelists from previous publications with the *AddModuleScore* function. Other 10X and Smart-seq datasets were mapped to the 10X mesenchymal atlas using *FindTransferAnchors* and *MapQuery* functions. To align stromal nomenclature, datasets from Wolock et al., 2019 [GSE132151][42] and Tikhonova et al., 2019 [GSE108892][19] were also analyzed in this study. For GSE132151, counts and metadata were downloaded and imported into Seurat, and processed as for GSE128423, except that cell annotations from the original study were already available in the metadata. For GSE108892, the count tables and metadata for 5 samples corresponding to steady state and the controls for the 5FU experiments were downloaded (GSM2915575, GSM2915576, GSM2915577, GSM2915578, GSM2915579) and merged into a single Seurat object. This object was processed as for GSE128423, except that the original annotation of cells was available in the author metadata. All these

datasets were used for mapping between other datasets using the *FindTransferAnchors* and *MapQuery* functions.

Spearman correlations

The *clustify* function in the R package ClustifyR was used to generate spearman correlations for gene expression between Smart-seq stromal populations, 10X stroma map, or 10X mesenchymal subclusters and published bulk RNA sequencing or microarray data, using the top 2,000 highly variable genes from the scRNA-seq datasets. For bulk RNA sequencing data from Asada et al., 2017 [GSE89811][14], Xu et al., 2018 [GSE104701][16], Helbling et al., 2019 [GSE133922][26], He et al., 2017 [GSE98587][50], and Tikhonova et al., 2019 [GSE108892][19], tables of normalized gene counts were downloaded directly and used as input for spearman correlations. For Mamidi et al., 2023 [GSE143249][51], Fastq files were downloaded, trimmed with TrimGalore, and aligned to the murine mm10 genome using Salmon to generate a normalized counts table. For microarray data contained in Greenbaum et al., 2013 [GSE43613][13], Worthley et al., 2015 [GSE57729][47], Yu et al., 2015 [GSE66042][49], and Ding et al., 2012 [GSE33158][11], raw data were downloaded and normalized using the *rma* function in the R package oligo[85]. Gene annotations were added using the *annotateEset* function (affycoretools package)[86] and the appropriate reference for the microarray, before rows corresponding to the same genes were collapsed with the *collapseRows* function (WGCNA package)[87]. For human stromal populations from Bandyopadhyay et al., 2024 [GSE253355][29], the analyzed Seurat object was downloaded directly from GEO, and normalized pseudobulk profiles were obtained using the *AggregateData* function in Seurat. Gene names in the murine Smart-seq and 10X stroma map were converted to human orthologs using the *getBM* function in the R package biomaRt[88]. Output from clustify was scaled between 0 and 1 across stromal populations or mesenchymal subclusters.

Stromal colony assays

For CFU-F assays, Endo MSC-S cells (15-300 cells) and mMPr/MPr (170-300 cells) were sorted directly into 6-well plates containing 1.5 ml αMEM supplemented with 10% FBS, 100 U/ml penicillin/100 μg/ml streptomycin, and 50 μM 2-mercaptoethanol. Cells were cultured under normal oxygen level for 11 days before staining with Giemsa-Wright to score colonies of 25 or more cells. CM or Endo MSC-L cells (100-300 cells) or (i)MSC-L (1,000 cells) were sorted directly into 6-well plates containing 1.5 mL of DMEM supplemented with 20% FBS, 100 U/ml penicillin/100 μg/ml streptomycin, 50 μM 2-mercaptoethanol, and 10 μM ROCK inhibitor (iROCK, Tocris, Y-27632). Cells were cultured in hypoxic conditions (5% O_2) for 8 days before staining with Giemsa-Wright to score colonies of 25 or more cells. For osteoblastic differentiation, αMEM or DMEM based medium were further supplemented with 3 mM β-glycerol phosphate (Sigma G9891) and 50 μg/mL ascorbic acid-2-phosphate (Sigma A8950). To stain for alkaline phosphatase, MSC-S cells (15-300 cells), mMPr/MPr (170-300 cells), or MSC-L cells (100-300 cells) were sorted directly into 6-well plates containing 1.5 ml of their respective culture medium, which was replaced with osteoblastic differentiation medium 2 days later. The cells were cultured for a further 9 days (until day 11 after isolation) and fixed with 1:10 phosphate-buffered formalin (Fisher scientific, 23-245-684) for 10 minutes. The cells were stained for 1 hour with 0.1 M Tris-HCl, pH 8.3 containing 0.6 mg/ml Fast Red Violet LB Salt (Sigma, F3381), 0.1 mg/ml Naphthol AS-MX phosphate (Sigma, N4875), and 0.4% N,N-dimethylformamide before counting CFU-ALP colonies of at least 25 cells. For Von Kossa staining, the cell culture was maintained in osteoblastic differentiation medium until day 23, fixed in phosphate-buffered formalin for 10 minutes, and then stained with 1% silver nitrate solution (Electron Microscopy Sciences 26212-01) for 20 minutes with light exposure, and rinsed with sodium thiosulfate (Ricca Chemical R7866500). For chondrocyte differentiation, 300 $CD24^+$ or $CD24^-$ mMPr/MPr were sorted directly into 6-well plates containing 1.5 ml MSC-S culture medium and cultured

for 1 week, replacing the medium every 2-3 days. The medium was then replaced with StemPro Chondrogenesis Differentiation Kit solution (ThermoFisher Scientific, A1007101), and the cells were cultured for another 2 weeks with medium changes every 3 days. For Toluidine blue staining, the cells were washed with PBS and fixed with phosphate-buffered formalin (Epredia, 9400-1) for 10 minutes. The cells were washed with PBS and stained with 0.04% Toluidine blue (Sigma-Aldrich, 89640-5G) in 0.1 M sodium acetate buffer (Fisher Scientific, BP333-500) at pH 4 for 10 minutes. The cells were then washed with MilliQ water and stained with 0.1% Fast Green (Fisher Scientific, BP123-10) in MilliQ water for 3 minutes. After another wash with MilliQ water, images were obtained with a microscope (Nikon Eclipse Ts2 or Keyence BZ-X810) with a 4X objective lens and software NIS-Elements D (5.11.00). ImagesJ (1.53v) was used to quantify the mean gray value for all the images.

Complete blood cell counts

Blood was collected by cardiac puncture after euthanasia and immediately transferred into EDTA-coated tubes (Greiner Bio-One, 0.5 ml). Complete blood cell counts were performed with an Oxford Science Genesis analyzer.

Micro-computed tomography

Computed tomographic (CT) scans were performed on a vivaCT80 microCT (Scanco Medical; 55 kV X-ray energy, 145 µA current, 10.4 µm isotropic voxel size, 250 ms integration time) and bone morphometric analyses were performed. Trabecular tissue volume, mineralized bone volume and trabecular bone parameters were determined from 97 slices, starting 0.2 mm proximal from the bottom edge of the growth plate using segmentation values of 0.8/1/360, which correspond to 584.3185 mg hydroxyapatite per cm^3.

Bone drilling injury model

3-month-old *Lepr-Cre*$^{+/-}$*:ROSA26-LSL-tdTomato*$^{+/-}$*:Col2.3*$^{GFP/+}$ mice were injected with PBS or 10mg/kg pIC every other day for 8 injections. The bone drilling was performed the day after the last injection. Under anesthesia induced with isoflurane, an incision was made into the skin over the medial aspect of the proximal tibia to expose the bone. A hole of diameter 0.6-0.9mm was then drilled using a hand-held drill (Yakamoz 0.3mm - 4mm Micro Aluminum Portable Handheld Drill) until the marrow cavity was exposed. The muscle and skin were closed using wound clips. The mice were euthanized 2 weeks after the surgery. The tibia was collected, cleaned, and frozen in OCT at -80°C. The tibia samples were sectioned using a Leica CM3050S cryostat with tungsten carbide blade until the cross section of the drilled hole could be seen clearly. The sample were then left at room temperature for the OCT to melt, washed with PBS, and stained with DAPI (5 μg/ml) for 10 minutes. The images were taken from the sectioned bones with a SP8 inverted confocal microscope (Leica) with a 10X objective lens using Leica Application Suite X. Two or three 2 μm z-stacks were acquired, and final images are maximum intensity projections.

Whole mount preparation

After mice were euthanized, the femurs were collected, cleaned, and frozen in OCT at -80°C. The femurs were sectioned longitudinally using a Leica CM3050S cryostat with tungsten carbide blade until the bone marrow cavity was maximally exposed. The samples were then left at room temperature for the OCT to melt, washed with PBS, and fixed with 4% PFA overnight. The bones were washed with PBS, blocked with 10% donkey serum (Millipore S30-100ML) in PBS for 2 hours, and stained with goat anti-LepR-biotin (R&D, BAF497; 1:100) in 10% donkey serum overnight. The bones were then washed with PBS and stained with donkey anti-goat A594 (Invitrogen A-11058; 1:100) for 2 hours. After another wash with PBS, the bones were blocked with rat IgG (Sigma-Aldrich I8015-50MG; 1:50) for 2 hours and stained

with anti-PDGFRα-AF488 (R&D FAB1062G; 1:100), anti-Sca-1-AF647 (Biolegend 108118; 1:100), and DAPI (5 μg/ml) overnight. The bones were washed again with PBS after the staining, and images were acquired with a SP8 inverted confocal microscope (Leica) with a 10X objective lens using Leica Application Suite X. Two μm z-stacks across 50 μm total thickness were acquired, and final images are maximum intensity projections.

BM section imaging

Bones for hematoxylin and eosin (H&E) staining were processed by the Molecular Pathology core facility at Columbia University Irving Medical Center. For other imaging, whole femurs were dissected, placed in plastic mounts containing OCT, and immediately frozen at -80°C. Sections of 7 μm thickness were cut using a Leica CM3050S cryostat with tungsten carbide blade and transferred to glass slides using a CryoJane tape transfer system (Leica). Slides were dried for 20 minutes at room temperature then fixed in acetone at -20°C for 10 minutes. When dry, a circle was drawn around tissue sections using a PAP pen (Ted Pella) before washing three times for 5 minutes with PBS. For imaging monocytes, slides were blocked with 10% donkey serum in PBS for 1 hour at room temperature and then stained with goat anti-mouse LepR-biotin primary antibody (1:100 dilution in PBS with 10% goat serum) for 2 hours at room temperature. After this, slides were washed three times for 5 minutes with PBS, then stained with donkey anti-goat IgG AF594 secondary antibody (Invitrogen, 1:100 dilution), anti-Ly6C – APC (BioLegend, 128016, 1:100), anti-CD11b – AF488 (BioLegend 101217, 1:100 dilution), and DAPI (5 μg/ml) in PBS with 10% donkey serum for 90 minutes at room temperature. Slides were washed as before then mounted with ProLong Glass Antifade Mountant (ThermoFisher Scientific) and a coverslip was applied. Images were acquired with a SP8 inverted confocal microscope (Leica) with a 20X objective lens using Leica Application Suite X. A z-stack of 1 μm was acquired across sections, and final images are maximum intensity projections.

Image analysis

For monocyte sections, cell segmentation was performed with Cellpose (v1.0.2), using model 'cyto3' and the Ly6C channel with cell diameter 5 pixels. Monocytes were defined by co-expression of CD11b and Ly6C among segmented cells. For whole mounts, images were analyzed with Imaris (Oxford Instruments, v10.2). The cortical bone volume was excluded from analysis by selecting a region of interest inside the bone cavity. The endosteal region was defined by creating a region of interest by hand tracing ~15 μm inside the bone surface. The spot function was used to segment individual nuclei, and automatic thresholds based on mean intensity in each section were applied for staining markers. MSC-L were defined as cells co-expressing PDGFRα and LepR but not expressing Sca-1, whereas MSC-S were identified as cells co-expressing PDGFRα and Sca-1 but not expressing LepR. The Imaris plugin *vantage* was used to count MSC-L, MSC-S, and total DAPI cells in endosteal and central marrow regions.

BM fluid isolation and assays

To isolate BM fluid, 2 tibiae from each mouse were flushed 15 times each with the same 350 μl of HBSS with 2% FBS into 1.5 ml tubes. Tubes were then centrifuged at 1,000 *g* for 10 minutes, and the supernatant was removed into new tubes and frozen at -80°C. Concentrations of chemokines were measured in BM fluid using the LEGENDplex mouse proinflammatory chemokine panel (13plex, BioLegend) with a V bottom plate according to the kit manual. Standards were diluted in matrix B, and samples were diluted 1:2 in assay buffer. Data were acquired with a Novocyte Penteon (Agilent).

MSC-L secretome analysis

To measure chemokine production from MSC-L, 4,000 MSC-L were sorted by FACS from mice injected with PBS or pIC for 3 days. Cells were sorted into 100 μl of medium composed of 50% αMEM and 50% DMEM medium with 10% FBS, 100 U/ml penicillin and 100 μg/ml streptomycin, and 10 μM

ROCK inhibitor in V-bottom tissue culture coated plates. After incubation at 37°C and 5% CO_2 in a water jacket incubator for 24 hours, plates were centrifuged and supernatant was stored at -80°C until chemokines were measured using the LEGENDplex mouse proinflammatory chemokine panel (13plex, BioLegend) with a V bottom plate according to the kit manual. Standards were diluted in matrix B, and samples were not diluted. Data were acquired with a Novocyte Penteon (Agilent).

Transwell assays

To evaluate chemoattractant activity of BM fluid from PBS or 3 day-pIC-exposed mice, 50 µl of BM fluid was diluted with 750 µl of complete IMDM medium (containing 5% FBS, 100 U/ml penicillin and 100 µg/ml streptomycin, 1X non-essential amino acids, 1 mM sodium pyruvate, 1X GlutaMAX, and 50 µM 2-mercaptoethanol), and placed in the bottom of a 24 well plate. A transwell insert (6.5 mm diameter, 5 µm pores) was then placed into the well. Whole BM was obtained from *Ifnar-/-* mice by flushing two femurs with a 3 ml syringe and a 21G needle and performing ACK RBC lysis. 10^6 whole BM cells were placed into the top of the transwell insert in a volume of 100 µl of complete IMDM medium, and the plate was incubated at 37°C with 5% CO_2 for 2 hours. After this, the transwell insert was removed and the medium in the well was re-suspended and transferred to 1.5 ml tubes, centrifuged, and stained with an antibody mixture of Ly6G-FITC (BioLegend, 127606, 1:400), Ly6C-APC/Cy7 (BioLegend, 128026, 1:400), CD8-AF700 (BD, 557959, 1:400), B220-BV421 (BioLegend, 103240, 1:800), CD4-BV510 (BioLegend, 100553, 1:400), CD11b-PE/Cy7 (Invitrogen, 25-0112-82, 1:800), CD115-PE (BioLegend, 135506, 1:100), CD3-APC (BioLegend, 100220, 1:100), and NK1.1-PE/Cy5 (BioLegend, 108716, 1:400) for 30 minutes on ice. Cells were then washed and re-suspended in HBSS with 2% FBS with 1 µg/ml propidium iodide before acquisition on a Novocyte Quanteon or Penteon (Agilent).

qRT-PCR analyses

Approximately 10,000 cells per population were sorted directly by FACS into RLT plus lysis buffer (Qiagen) with 2-mercaptoethanol and stored at -80 °C until purification with a RNeasy Plus Micro Kit (Qiagen) according to the manufacturer's protocol. Following column purification, RNA was immediately reverse transcribed using a SuperScriptIII kit with random hexamers (ThermoFisher Scientific). RT-qPCR runs were performed on a QuantStudio 7 Flex Real-Time PCR system (Applied Biosystems) using SYBR Green reagents (Bio-Rad), the cDNA equivalent of 200 cells per reaction, and triplicate technical measurements per biological repeat. Cycle threshold values were normalized to *Actb*.

Statistics and reproducibility

All experiments were repeated as indicated; n indicates the numbers of independent biological repeats. Data are expressed as the mean ± s.d. unless otherwise indicated. Mice used for treatment were assigned to experimental groups based on age and genotype, randomized with respect to sex, and samples were alternated whenever possible. Data collection and analysis were not performed blind to the conditions of the experiments. Data were processed using Microsoft Excel (v.16), and statistical significance was evaluated using GraphPad Prism (v.10). Figures were made using GraphPad Prism. Data distribution was assumed to be normal, but this was not formally tested.

Additional references for methods

71. Andrews S (2010) FastQC: a quality control tool for high throughput sequence data. http://www.bioinformatics.babraham.ac.uk/projects/fastqc.

72. Krueger F (2024) TrimGalore. https://github.com/FelixKrueger/TrimGalore.

73. Patro R, Duggal G, Love MI, Irizarry RA, Kingsford C (2017) Salmon: fast and bias-aware quantification of transcript expression using dual-phase inference. *Nat Methods* **14**:417-419.

74. Hao Y, Hao S, Andersen-Nissen E, Mauck WM, Zheng S, Butler A, Lee MJ, Wilk AJ, Darby C, Zager M, Hoffman P, Steockius M, Papalexi E, Mimitou EP, Jain J, Srivastava A, Stuart T, Fleming LM, Yeung B, Rogers AJ, McElrath JM, Blish CA, Gottardo R, Smibert P, Satija R (2021) Integrated analysis of multimodel single-cell data. *Cell* **184**:3573-3587.

75. Hao Y, Stuart T, Kowalski MH, Choudhary S, Hoffman P, Hartman A, Srivastava A, Molla G, Madad S, Fernandez-Granda C, Satija R (2024) Dictionary learning for integrative, multimodel and scalable single-cell analysis. *Nat Biotechnol* **42**:293-304.

76. Young MD, Behjati S (2020) SoupX removes ambient RNA contamination from droplet-based single-cell RNA sequencing data. *GigaScience* **9**:giaa151.

77. Fu R, Gillen AE, Sheridan RM, Tian C, Daya M, Hao Y, Hesselberth JR, Riemondy KA (2020) clustifyr: an R package for automated single-cell RNA sequencing cluster classification. *F1000Res* **9**:223.

78. Yu G, Wang LG, Han Y, He QY (2012) clusterProfiler: an R package for comparing biological themes among gene clusters. *OMICS* **16**:284-287.

79. Wu T, Hu E, Xu S, Chen M, Guo P, Dai Z, Feng T, Zhou L, Tang W, Zhan L, Fu X, Liu S, Bo X, Yu G (2021) clusterProfiler 4.0: a universal enrichment tool for interpreting omics data. *Innovation* **2**:100141.

80. Moon KR, van Dijk D, Wang Z, Gigante S, Burkhardt DB, Chen WS, Yim K, van den Elzen A, Hirn MJ, Coifman RR, Ivanova NB, Wolf G (2019) Visualizing structure and transitions in high-dimensional biological data. *Nat Biotechnol* **37**:1482-1492.

81. Street K, Risso D, Fletcher RB, Das D, Ngai J, Yosef N, Purdom E, Dudoit S (2018) Slingshot: lineage and pseudotime inference for single-cell transcriptomics. *BMC Genomics* **19**:477.

82. Heinz S, Benner C, Spann N, Bertolino E, Lin YC, Laslo P, Cheng JX, Murre C, Singh H, Glass CK (2010) Simple combinations of lineage-determining transcription factors prime cis-regulatory elements required for macrophage and B cell identities. *Mol Cell* **38**:576-589.

83. Zhang Z, Luo D, Zhong X, Choi JH, Ma Y, Wang S, Mahrt E, Guo W, Stawiski EW, Modrusan Z, Seshagiri S, Kapur P, Hon GC, Brugarolas J, Wang T (2019) SCINA: a semi-supervised subtyping algorithm of single cells and bulk samples. *Genes* **10**:531.

84. Trapnell C, Cacchiarelli D, Grimsby J, Pokharel P, Li S, Morse M, Lennon NJ, Livak KJ, Mikkelsen TS, Rinn JL (2014) The dynamics and regulators of cell fate decisions are revealed by pseudotemporal ordering of single cells. *Nat Biotechnol* **32**:381-386.

85. Carvalho BS, Irizarry RA (2010) A framework for oligonucleotide microarray preprocessing. *Bioinformatics* **26**:2363-2367.

86. MacDonald JW (2024) affycoretools: Functions useful for those doing repetitive analyses with Affymetrix GeneChips. R package version 1.76.0.

87. Langfelder P, Horvath S (2008) WGCNA: an R package for weighted correlation network analysis. *BMC Bioinformatics* **9**:559.

88. Durinck S, Spellman P, Birney E, Huber W (2009) Mapping identifiers for the integration of genomic datasets with the R/Bioconductor package biomaRt. *Nat Protoc* **4**:1184-1191.

Supplemental Tables

Supplemental Table 1. Distribution of stromal and hematopoietic cells in bone marrow spatial compartments. Distribution of cells in Louvain clusters in 10X stromal map shown in Fig. 1c, with origin of cells in either central marrow or endosteal samples. Top 50 most significantly upregulated genes in each cluster compared to all other clusters.

(separate Excel file)

Supplemental Table 2. Key gene markers in Smart-seq populations. Top 100 most significantly upregulated genes in each stromal population compared to all other cell types.

(separate Excel file)

Supplemental Table 3. Gene signatures used for annotation of MSCs in 10X mesenchymal atlas

Cell	$LepR^+$ MSC	Sca-1^+ MSC
Genes	*Lepr*	*Ly6a*
	Ibsp	*Cd34*
	Mgp	*Thy1*
	Ogn	*Mfap5*
	Cxcl12	*Gsn*
		Clec3b

Genes used for annotation of mesenchymal stromal cell (MSC) populations in 10X mesenchymal atlas.

Supplemental Figures

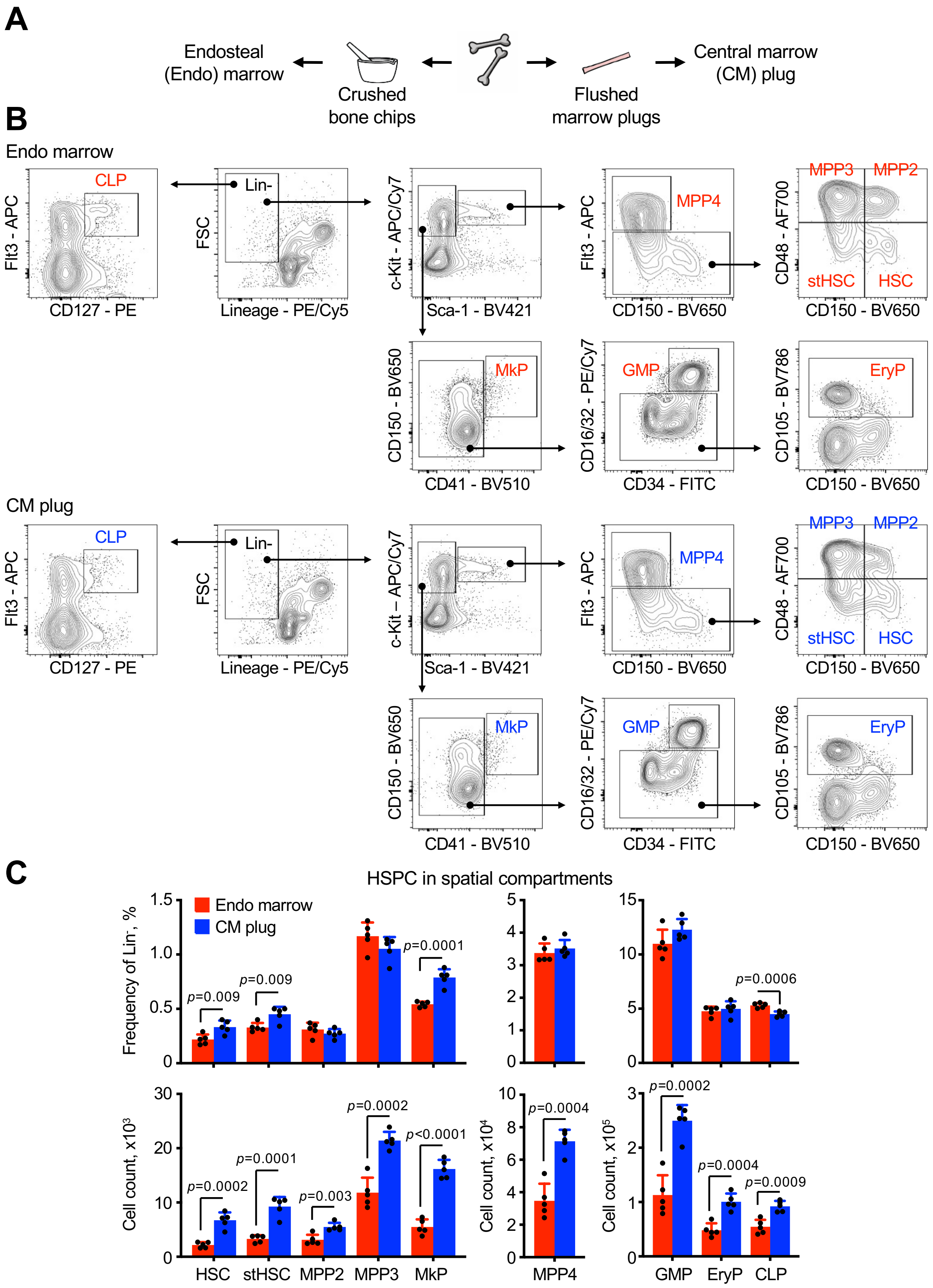
A
Endosteal (Endo) marrow
Crushed bone chips
Flushed marrow plugs
Central marrow (CM) plug
B
Endo marrow
CLP
Lin-
MPP4
MPP3
MPP2
stHSC
HSC
MkP
GMP
EryP
Flt3 - APC
CD127 - PE
FSC
Lineage - PE/Cy5
c-Kit - APC/Cy7
Sca-1 - BV421
CD150 - BV650
CD48 - AF700
CD41 - BV510
CD16/32 - PE/Cy7
CD34 - FITC
CD105 - BV786
CM plug
C
HSPC in spatial compartments
Endo marrow
CM plug
Frequency of Lin⁻, %
Cell count, x10³
Cell count, x10⁴
Cell count, x10⁵
p=0.009
p=0.009
p=0.0001
p=0.0006
p=0.0002
p=0.0001
p=0.003
p=0.0002
p<0.0001
p=0.0004
p=0.0002
p=0.0004
p=0.0009
HSC
stHSC
MPP2
MPP3
MkP
MPP4
GMP
EryP
CLP

Supplemental Figure 1. Spatial compartmentalization of stromal and hematopoietic cells. A, Scheme showing the isolation of CM plug from flushed bones and Endo marrow from crushed bones. **B**, gating scheme for the indicated HSPC populations in Endo marrow (top) and CM plug (bottom). HSC: hematopoietic stem cell; stHSC: short-term HSC; MPP2/3/4: multipotent progenitor 1/2/3; MkP: megakaryocyte progenitor; CLP: common lymphoid progenitor; GMP: granulocyte macrophage progenitor; EryP: erythroid progenitor. **C**, Frequency among lineage negative BM cells (top) and absolute numbers (bottom) of indicated hematopoietic stem and progenitor cells (HSPC) in central marrow (CM) and endosteal (Endo) fractions. Data in are means ± S.D. with points showing values for individual mice. *P. values*, derived from two-way ANOVA with Sidak's *post hoc* test.

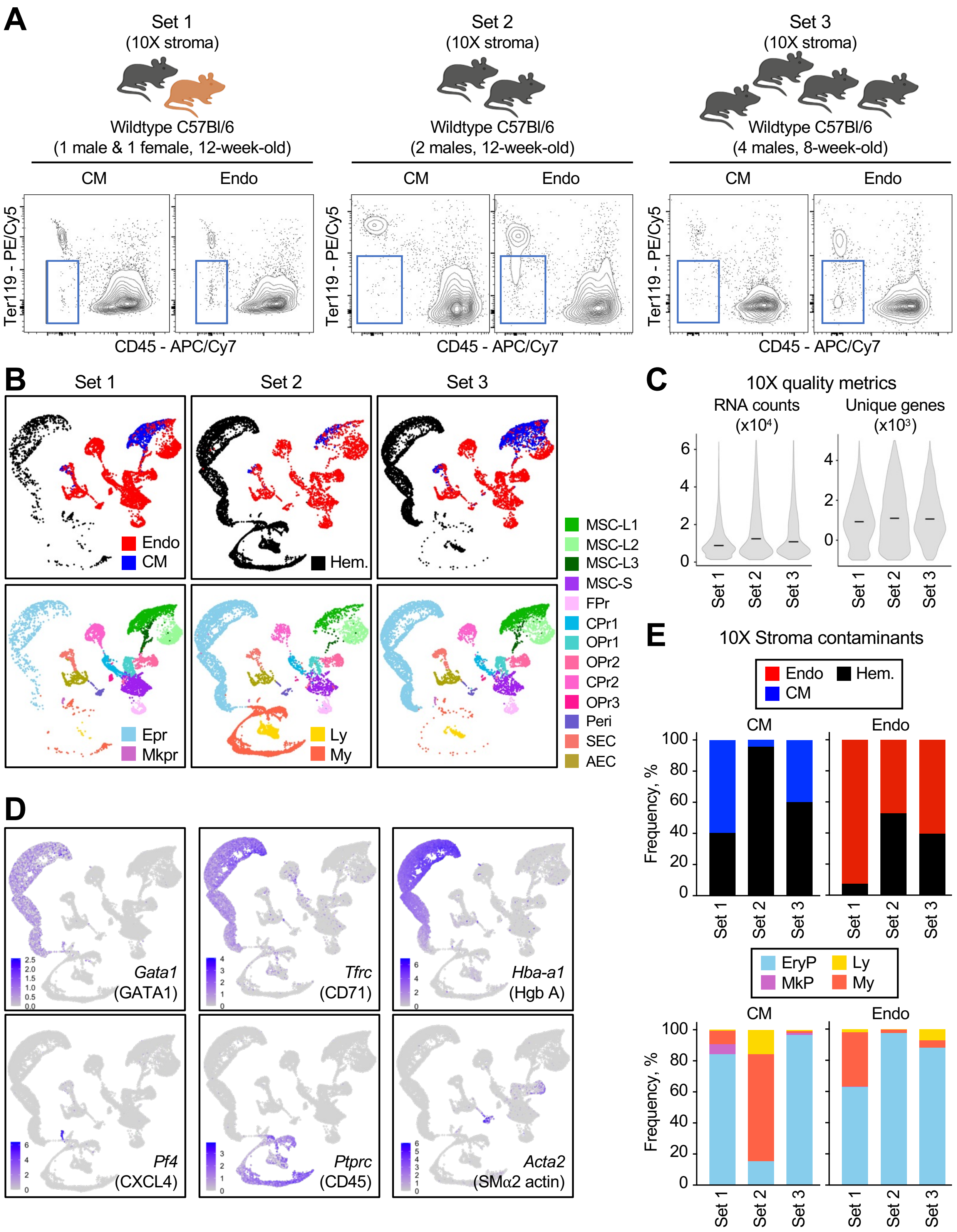
A
Set 1
(10X stroma)
Wildtype C57Bl/6
(1 male & 1 female, 12-week-old)
Set 2
(10X stroma)
Wildtype C57Bl/6
(2 males, 12-week-old)
Set 3
(10X stroma)
Wildtype C57Bl/6
(4 males, 8-week-old)
CM
Endo
Ter119 - PE/Cy5
CD45 - APC/Cy7
B
Set 1
Set 2
Set 3
Endo
CM
Hem.
Epr
Mkpr
Ly
My
MSC-L1
MSC-L2
MSC-L3
MSC-S
FPr
CPr1
OPr1
OPr2
CPr2
OPr3
Peri
SEC
AEC
C
10X quality metrics
RNA counts (x10⁴)
Unique genes (x10³)
D
Gata1 (GATA1)
Tfrc (CD71)
Hba-a1 (Hgb A)
Pf4 (CXCL4)
Ptprc (CD45)
Acta2 (SMα2 actin)
E
10X Stroma contaminants
Endo
Hem.
CM
Frequency, %
EryP
Ly
MkP
My

Supplemental Figure 2. Generation of integrated stromal map. **A**, Scheme showing independent biological replicates used for isolation of central marrow (CM) and endosteal (Endo) stromal preparation for 10X single cell RNA sequencing (scRNA-seq), with flow cytometric images to show gates used for sorting $CD45^-$/$Ter119^-$ live cells for scRNA-seq. **B**, UMAP dimension reductions showing origin of cells in either CM or Endo samples, with contaminating hematopoietic cells (Hem.) (top) and annotation of stromal cell populations corresponding to **Figure 1C** (bottom). MSC-L: leptin receptor expressing mesenchymal stromal cell; MSC-S: Sca-1 expressing MSC; FPr: fibroblast progenitor; CPr: chrondroblast progenitor; OPr: osteoblast progenitor; Peri: pericyte; SEC: sinusoidal endothelial cell; AEC: arterial endothelial cell. **C**, Violin plots showing RNA counts and number of unique genes observed in each replicate dataset, with medians. **D**, Feature plots showing expression of indicated genes in the 10X stroma dataset. **E**, Barplots showing composition of contaminating hematopoietic (Hem.) cell types in each replicate dataset. EryP: erythroid progenitor; MkP: megakaryocyte progenitor; Ly: lymphocyte; My: myeloid lineage cells.

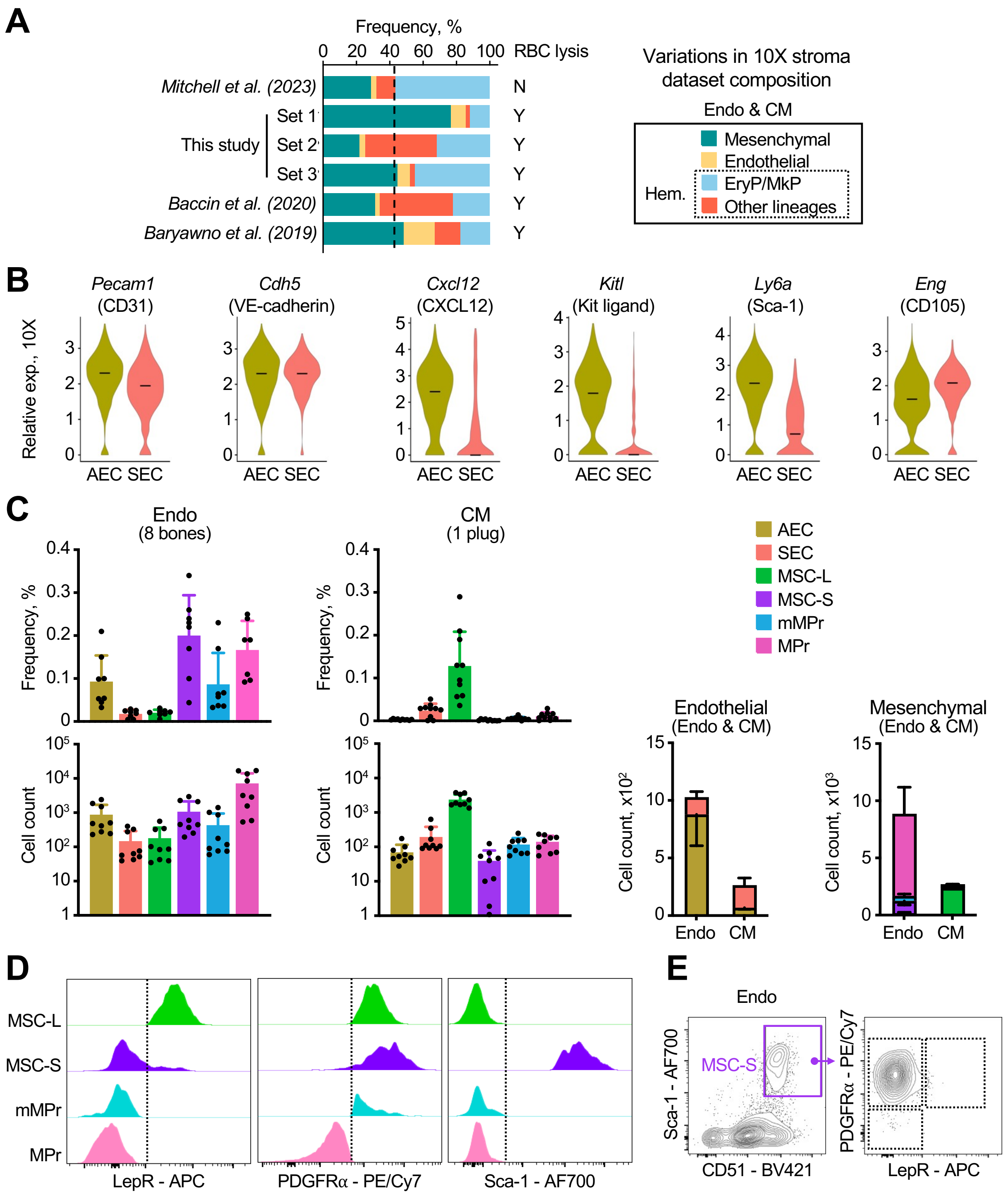
A
Frequency, %
RBC lysis
Mitchell et al. (2023)
This study
Set 1
Set 2
Set 3
Baccin et al. (2020)
Baryawno et al. (2019)
Variations in 10X stroma dataset composition
Endo & CM
Mesenchymal
Endothelial
Hem.
EryP/MkP
Other lineages
B
Pecam1 (CD31)
Cdh5 (VE-cadherin)
Cxcl12 (CXCL12)
Kitl (Kit ligand)
Ly6a (Sca-1)
Eng (CD105)
Relative exp., 10X
AEC SEC
C
Endo (8 bones)
CM (1 plug)
Frequency, %
Cell count
AEC
SEC
MSC-L
MSC-S
mMPr
MPr
Endothelial (Endo & CM)
Mesenchymal (Endo & CM)
Cell count, x10²
Cell count, x10³
Endo CM
D
MSC-L
MSC-S
mMPr
MPr
LepR - APC
PDGFRα - PE/Cy7
Sca-1 - AF700
E
Endo
MSC-S
Sca-1 - AF700
CD51 - BV421
PDGFRα - PE/Cy7
LepR - APC

Supplemental Figure 3. Characterization of stromal compartments. **A**, Frequency of indicated cell types in stroma scRNA-seq datasets from this study (Set 1-3), and from previously published datasets with (Y: yes) or without (N: no) red blood cell (RBC) lysis. Hem.: hematopoietic cells. **B**, Expression of surface marker genes associated with arterial endothelial cell (AEC) and sinusoidal endothelial cell (SEC) cluster identity in 10X stroma dataset. **C**, Frequency (top) and absolute number (bottom) of major stromal cell populations in CM and Endo preparations. MSC-L: $LepR^+$ mesenchymal stromal cell, MSC-S: $Sca\text{-}1^+$ mesenchymal stromal cell, (m)MPr: (multipotent) mesenchymal progenitor. **D**, Representative flow cytometric images showing expression of indicated markers in indicated stromal cell populations. (m)MPr: (multipotent) mesenchymal progenitor. **E**, Representative flow cytometric image showing lack of expression of leptin receptor in MSC-S from Endo preparation. Data in (C) are means ± S.D. with points showing values for individual mice. *P. values*, derived from Student's t test. Data in (B) are violin plots of relative expression (exp.) of 10X SCT transformed counts with median.

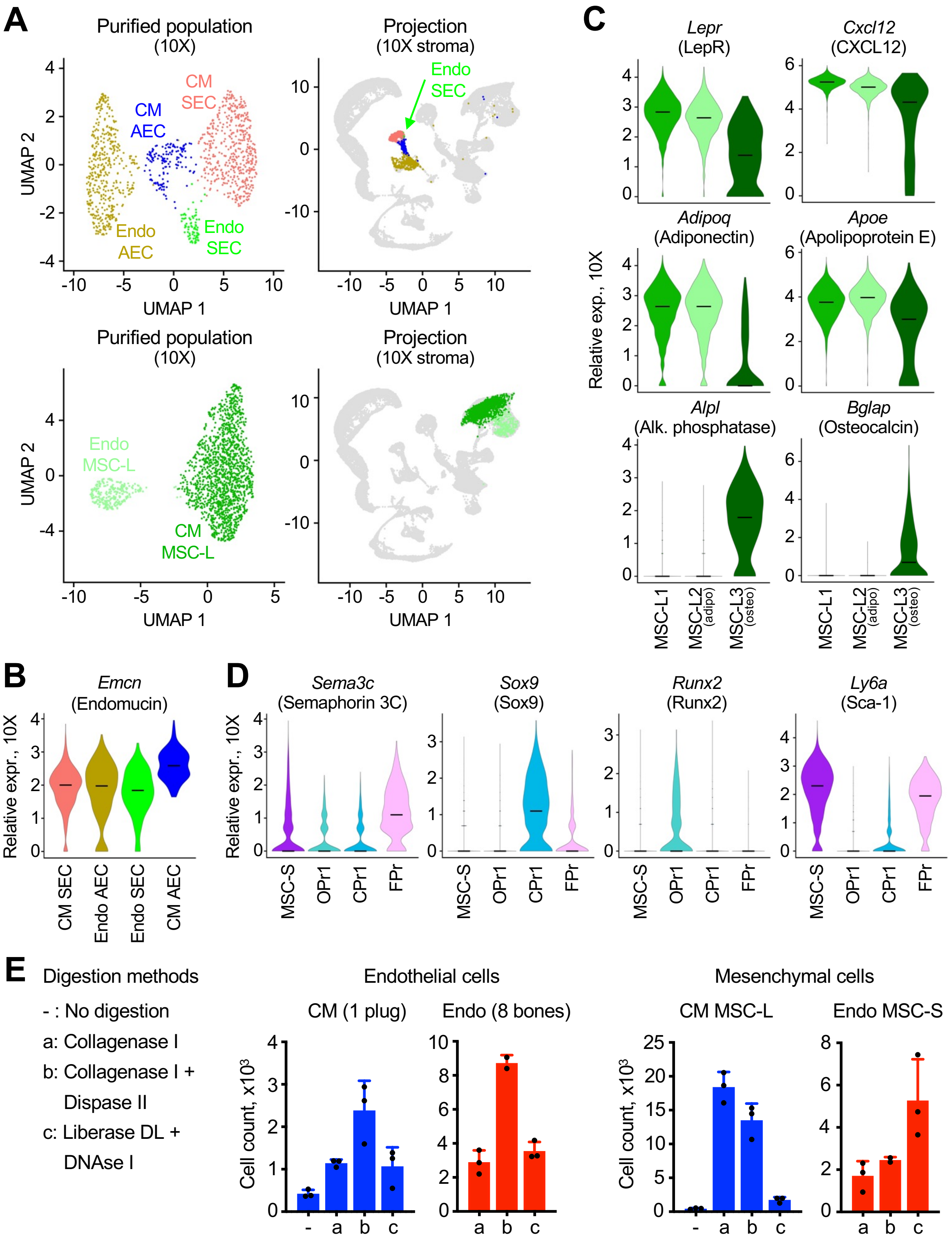
A
Purified population (10X)
Projection (10X stroma)
CM AEC
CM SEC
Endo AEC
Endo SEC
UMAP 1
UMAP 2
Endo MSC-L
CM MSC-L
B
Emcn (Endomucin)
Relative expr., 10X
CM SEC
Endo AEC
Endo SEC
CM AEC
C
Lepr (LepR)
Cxcl12 (CXCL12)
Adipoq (Adiponectin)
Apoe (Apolipoprotein E)
Alpl (Alk. phosphatase)
Bglap (Osteocalcin)
Relative exp., 10X
MSC-L1
MSC-L2 (adipo)
MSC-L3 (osteo)
D
Sema3c (Semaphorin 3C)
Sox9 (Sox9)
Runx2 (Runx2)
Ly6a (Sca-1)
MSC-S
OPr1
CPr1
FPr
E
Digestion methods
- : No digestion
a: Collagenase I
b: Collagenase I + Dispase II
c: Liberase DL + DNAse I
Endothelial cells
CM (1 plug)
Endo (8 bones)
Mesenchymal cells
CM MSC-L
Endo MSC-S
Cell count, x10³

Supplemental Figure 4. Optimization of stromal isolation approach. **A**, UMAP dimension reduction (left) and projection onto 10X stroma dataset (right) of purified SEC/AEC (top) and MSC-L (bottom) isolated from both CM and Endo fractions and analyzed by 10X scRNA-seq. **B**, Differential expression of *Emcn* (endomucin) gene in purified CM and Endo SEC/AEC. **C**, Expression of signature genes associated with MSC-L cluster identity in 10X stroma dataset. Adipo: adipocyte bias, Osteo: osteoblast biased. **D**, Expression of signature genes associated with MSC-S and mesenchymal progenitor cluster identity in 10X stroma dataset. OPr: osteoprogenitor, CPr: chrondroblast progenitor, FPr: fibroblast progenitor. **E**, Quantification of indicated stromal cell populations isolated from CM and Endo preparations using no digestion or the indicated digestion protocols (a-c). Data in (B), (C), and (D) are violin plots of relative expression (exp.) of 10X SCT transformed counts with median. Data in (E) are means ± S.D. with points showing values for individual mice.

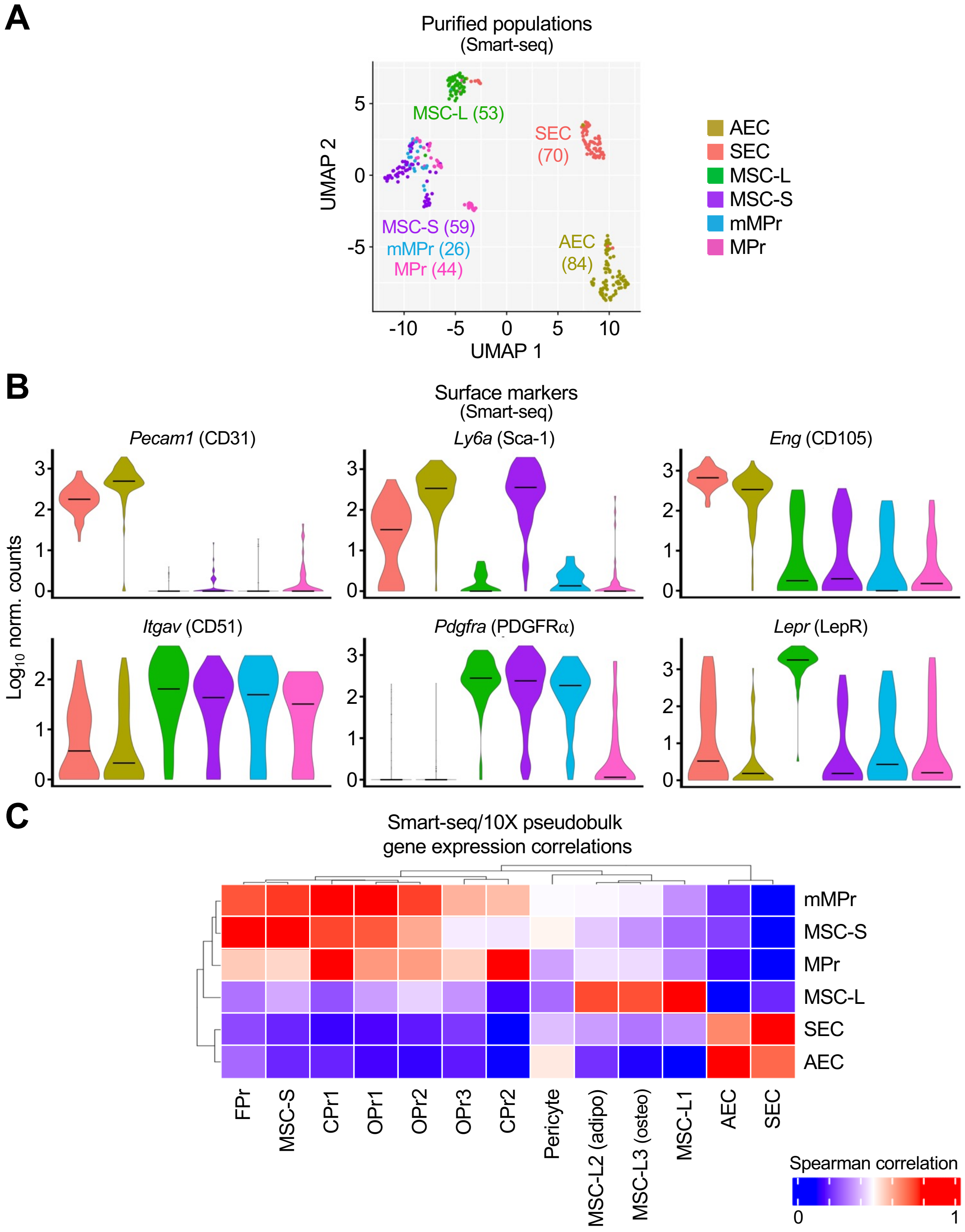
A
Purified populations
(Smart-seq)
MSC-L (53)
SEC
(70)
MSC-S (59)
mMPr (26)
MPr (44)
AEC
(84)
UMAP 2
UMAP 1
AEC
SEC
MSC-L
MSC-S
mMPr
MPr
B
Surface markers
(Smart-seq)
Pecam1 (CD31)
Ly6a (Sca-1)
Eng (CD105)
Itgav (CD51)
Pdgfra (PDGFRα)
Lepr (LepR)
Log10 norm. counts
C
Smart-seq/10X pseudobulk
gene expression correlations
mMPr
MSC-S
MPr
MSC-L
SEC
AEC
FPr
MSC-S
CPr1
OPr1
OPr2
OPr3
CPr2
Pericyte
MSC-L2 (adipo)
MSC-L3 (osteo)
MSC-L1
AEC
SEC
Spearman correlation
0
1

Supplemental Figure 5. Molecular characterization of major stromal populations. **A**, UMAP dimension reduction of purified major CM (SEC, MSC-L) and Endo (MSC-S, mMPr, MPr) stromal cells analyzed by Smart-seq and previously published in Mitchell et al. (2023)[4]. **B**, Expression of surface marker genes used to isolate major CM and Endo stromal cells for Smart-seq analyses. Data are violin plots of Log10 normalized (norm.) Smart-seq counts with median. **C**, Heatmap showing row-normalized Spearman correlations between indicated stromal populations isolated for Smart-seq analyses with pseudobulk profiles from clusters identified in the 10X stroma dataset.

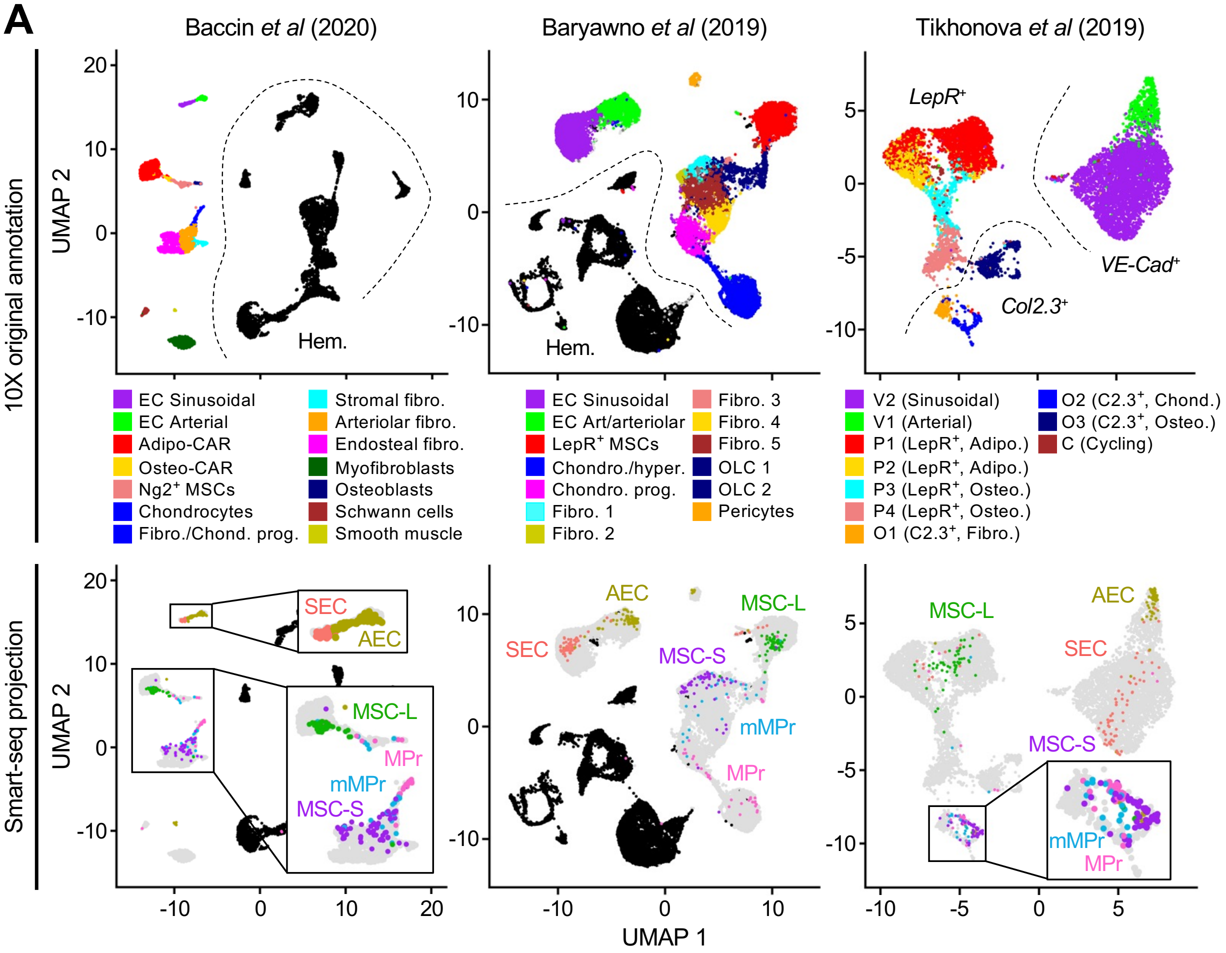

B

This study (Flow)	This study (10X)	Baccin *et al* (2020)	Baryawno *et al* (2019)	Tikhonova *et al* (2019)
MSC-L	MSC-L1<br>MSC-L2 (adipo)<br>MSC-L3 (osteo)	Adipo-CAR<br>Osteo-CAR	LepR$^+$ MSC	P1 (LepR$^+$, Adipo.)<br>P2 (LepR$^+$, Adipo.)
MSC-S	MSC-S<br>FPr	Endosteal fibroblast<br>Arteriolar fibroblast<br>Stromal fibroblast	Fibroblast 1<br>Fibroblast 2<br>Fibroblast 3	O2 (Col2.3$^+$, Chondro.)
mMPr	OPr1<br>CPr1	Fibro./Chondro. progen.	OLC 1<br>Fibroblast 5<br>Chondroblast progen.	O2 (Col2.3$^+$, Chondro.)
MPr	OPr2<br>OPr3<br>CPr2	Chondrocytes<br>Osteoblasts	Chondroblasts<br>Chondroblast progen.<br>OLC 1<br>OLC 2	O2 (Col2.3$^+$, Chondro.)
SEC	SEC	EC Sinusoidal	EC Sinusoidal	V2 (Sinusoidal)
AEC	AEC	EC Arterial	EC Arterial/Arteriolar	V1 (Arterial)

Supplemental Figure 6. Alignment of stromal nomenclature across studies. A, Projection of Smart-seq stromal cell transcriptomes onto the indicated previously published 10X stromal datasets, with original annotations derived from those studies. Hematopoietic cell (Hem.) contamination is shown in black and the fluorescence reporter mice lines used to identify stroma element is provided for Tikhonova et al. (2019)[19]. Of note, Baccin et al. (2020)[17] used undigested stromal preparation likely preserving isolation of Schwann and smooth muscle cells. **B**, Alignment of names for major stromal populations between this study and previous publications.

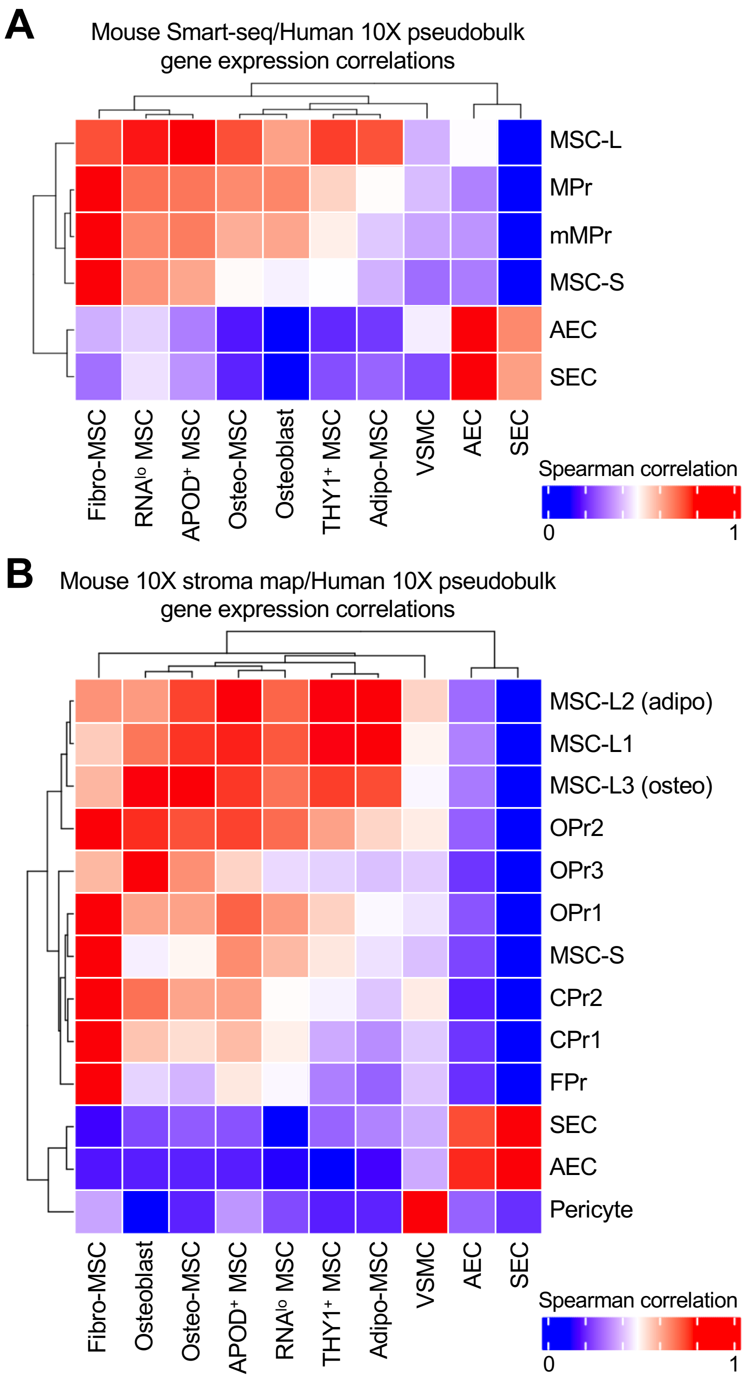
A
Mouse Smart-seq/Human 10X pseudobulk gene expression correlations
MSC-L
MPr
mMPr
MSC-S
AEC
SEC
Fibro-MSC
RNAlo MSC
APOD+ MSC
Osteo-MSC
Osteoblast
THY1+ MSC
Adipo-MSC
VSMC
AEC
SEC
Spearman correlation
0
1
B
Mouse 10X stroma map/Human 10X pseudobulk gene expression correlations
MSC-L2 (adipo)
MSC-L1
MSC-L3 (osteo)
OPr2
OPr3
OPr1
MSC-S
CPr2
CPr1
FPr
SEC
AEC
Pericyte
Fibro-MSC
Osteoblast
Osteo-MSC
APOD+ MSC
RNAlo MSC
THY1+ MSC
Adipo-MSC
VSMC
AEC
SEC
Spearman correlation
0
1

Supplemental Figure 7. Conservation of human and murine stromal cell types. A-B, Heatmaps showing row-normalized Spearman correlations between indicated stromal populations from Smart-seq (A) and 10X stroma map (B) with pseudobulk profiles from clusters identified in the human BM stroma atlas from Bandyopadhyay et al. (2024)[29].

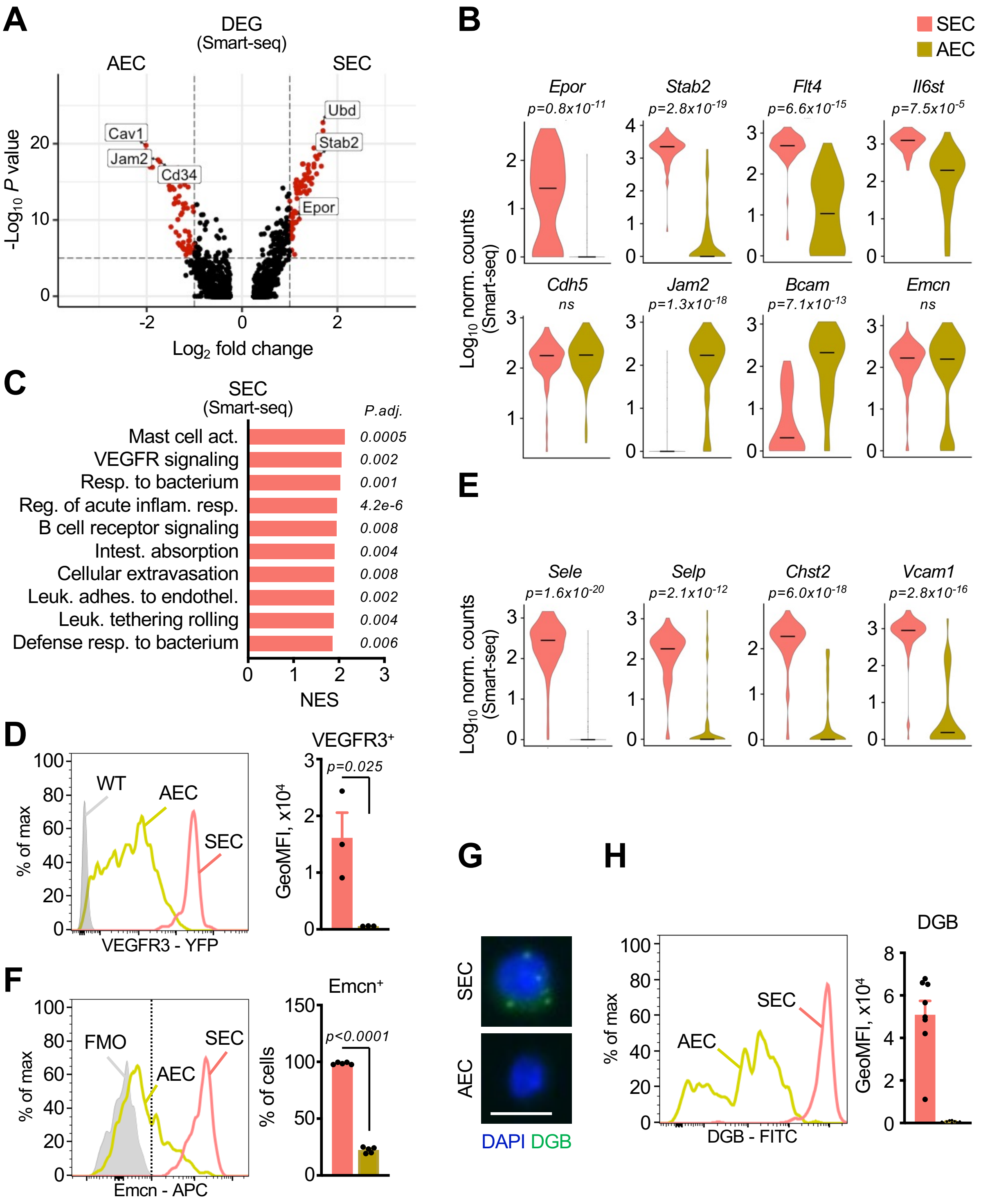

A
DEG
(Smart-seq)
AEC
SEC
Cav1
Jam2
Cd34
Ubd
Stab2
Epor
-Log10 P value
Log2 fold change
B
SEC
AEC
Epor
p=0.8x10-11
Stab2
p=2.8x10-19
Flt4
p=6.6x10-15
Il6st
p=7.5x10-5
Cdh5
ns
Jam2
p=1.3x10-18
Bcam
p=7.1x10-13
Emcn
ns
Log10 norm. counts
(Smart-seq)
C
SEC
(Smart-seq)
P.adj.
Mast cell act. 0.0005
VEGFR signaling 0.002
Resp. to bacterium 0.001
Reg. of acute inflam. resp. 4.2e-6
B cell receptor signaling 0.008
Intest. absorption 0.004
Cellular extravasation 0.008
Leuk. adhes. to endothel. 0.002
Leuk. tethering rolling 0.004
Defense resp. to bacterium 0.006
NES
D
% of max
WT
AEC
SEC
VEGFR3 - YFP
VEGFR3+
p=0.025
GeoMFI, x104
E
Sele
p=1.6x10-20
Selp
p=2.1x10-12
Chst2
p=6.0x10-18
Vcam1
p=2.8x10-16
Log10 norm. counts
(Smart-seq)
F
% of max
FMO
AEC
SEC
Emcn - APC
Emcn+
p<0.0001
% of cells
G
SEC
AEC
DAPI DGB
H
% of max
AEC
SEC
DGB - FITC
DGB
GeoMFI, x104

Supplemental Figure 8. Characterization of sinusoidal endothelial cells. A, Volcano plot showing significantly differentially expressed genes between arterial (AEC) and sinusoidal (SEC) endothelial cells profiled by plate-based Smart-seq scRNA-seq. Red points show genes with $\log_2$ fold change >|1| and adjusted p value $<10^{-5}$. **B**, Expression of signature genes associated with AEC and SEC identity. **C**, Geneset enrichment analysis (GSEA) of selectively enriched GO biological pathways in SEC compared to other stromal cells (NES: normalized enrichment score, P.adj.: adjusted *P values*). **D**, Representative flow cytometry plots (left) and quantification of geometric mean fluorescence intensity (GeoMFI, right) of VEGFR3 expression in SECs and AECs of *Vegfr3-Yfp* reporter mice. **E**, Violin plots showing expression of selected genes in SECs and AECs from Smart-seq scRNA-seq. **F**, Representative flow cytometry plots (left) and quantification (right) of the proportion of SECs and AECs stained with Endomucin antibody ($Emcn^+$ cells). **G-H**, Assessment of permeability of entothelial cells, with (G) representative images of dragon green bead (DEG) uptake (scale bar: 5 µm) and (H) representative flow cytometry plots (left) and quantification of geometric mean fluorescent intensity (GeoMFI, right) of DGB uptake. WT: wild type staining control included for the analyses of fluorescent reporter mice in (D), FMO: fluorescence minus one control included for the analyses of antibody staining in (F). Data in (B,E) are violin plots of Log10 normalized (norm.) Smart-seq counts; *P. values*, derived from Wilcoxon rank sum test. Data in (D,F,H) are means ± S.D. with points showing values for individual mice; *P. values*, derived from Student's t test. For (C), *P. values* are derived from permutation tests.

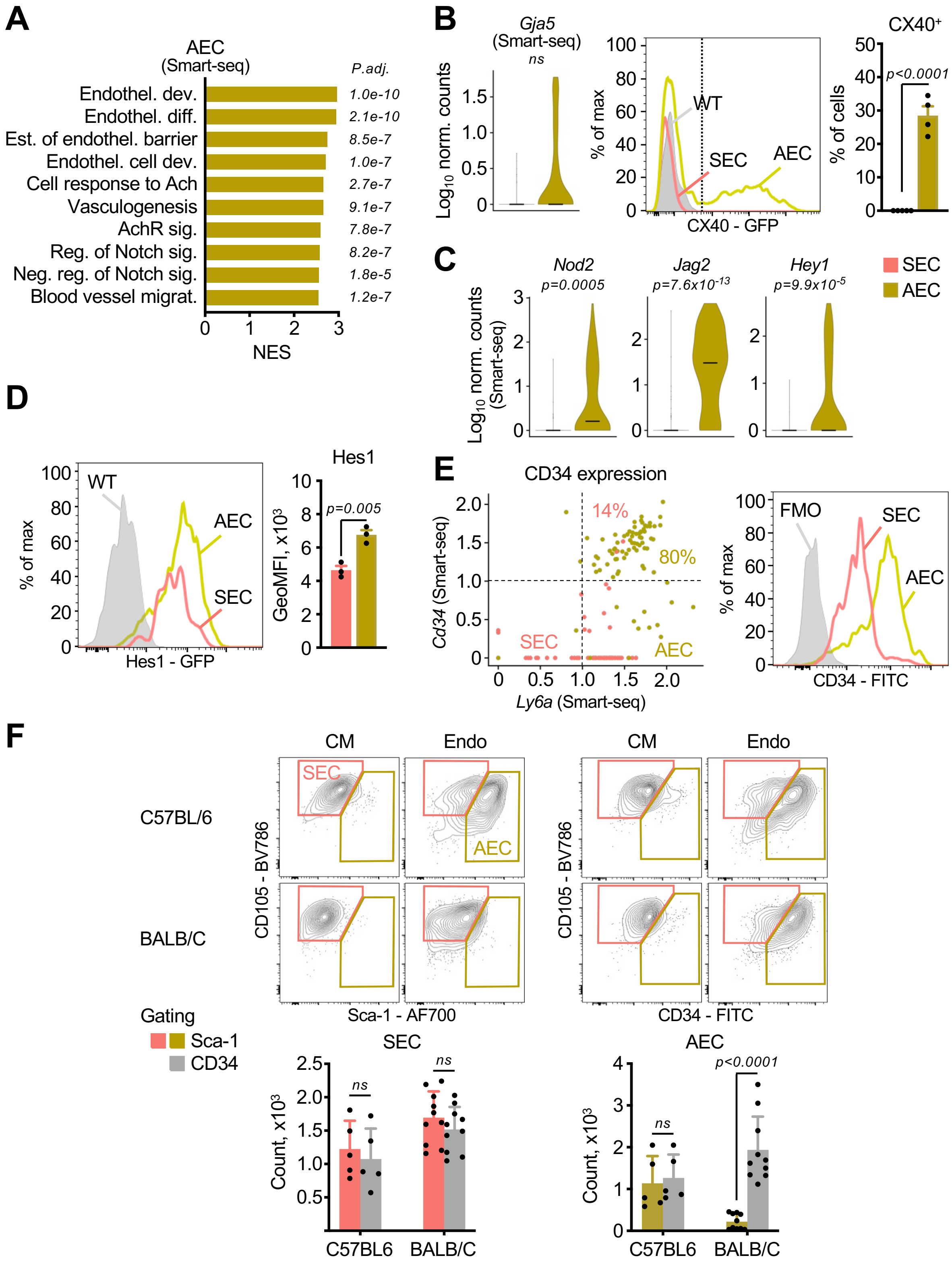

A
AEC (Smart-seq)
P.adj.
Endothel. dev. 1.0e-10
Endothel. diff. 2.1e-10
Est. of endothel. barrier 8.5e-7
Endothel. cell dev. 1.0e-7
Cell response to Ach 2.7e-7
Vasculogenesis 9.1e-7
AchR sig. 7.8e-7
Reg. of Notch sig. 8.2e-7
Neg. reg. of Notch sig. 1.8e-5
Blood vessel migrat. 1.2e-7
NES
B
Gja5 (Smart-seq) ns
Log10 norm. counts
% of max
WT
SEC
AEC
CX40 - GFP
CX40+
% of cells
p<0.0001
C
Nod2 p=0.0005
Jag2 p=7.6x10-13
Hey1 p=9.9x10-5
Log10 norm. counts (Smart-seq)
SEC
AEC
D
% of max
WT
AEC
SEC
Hes1 - GFP
Hes1
GeoMFI, x10³
p=0.005
E
CD34 expression
Cd34 (Smart-seq)
Ly6a (Smart-seq)
14%
80%
SEC
AEC
% of max
FMO
SEC
AEC
CD34 - FITC
F
CM
Endo
CM
Endo
C57BL/6
BALB/C
CD105 - BV786
Sca-1 - AF700
CD34 - FITC
Gating
Sca-1
CD34
SEC
AEC
Count, x10³
ns
p<0.0001
C57BL6
BALB/C

Supplemental Figure 9. Characterization of arterial endothelial cells. A, Geneset enrichment analysis (GSEA) of selectively enriched GO biological pathways in AEC compared to other stromal cells (NES: normalized enrichment score, P.adj.: adjusted *P values*). **B**, Expression of *Gja5* in Smart-seq SECs and AECs (left) with representative flow cytometry plots (middle) and quantification (right) of the proportion of SECs and AECs expressing connexin 40 (CX40) in *Cx40-Gfp* reporter mice. **C**, Violin plots showing expression of selected genes in SECs and AECs from Smart-seq scRNA-seq. **D**, Representative flow cytometry plots (left) and quantification of GeoMFI (right) of *Hes1*-GFP expression. **E**, Expression of CD34 (*Cd34*) in Smart-seq SECs and AECs compared to Sca-1 (*Ly6a*) (top) with representative flow cytometry plots of CD34 antibody staining in SECs and AECs (bottom). **F**, Representative flow cytometry plots showing identification (top) and quantification (bottom) of the number of SECs and AECs identified with either Sca-1 (left) or CD34 (right) antibody staining in C57BL/6 and BALB/C mice. WT: wild type staining control included for the analyses of fluorescent reporter mice in (D); FMO: fluorescence minus one control included for the analyses of antibody staining in (E). Data in (B/left,C) are violin plots of Log10 normalized (norm.) Smart-seq counts with median. Data in (B/right,D,F) are means ± S.D. with points showing values for individual mice. *P. values*, derived from Student's t test (B,D), two-way ANOVA with Sidak's *post hoc* test (F), or Wilcoxon rank sum test (C).

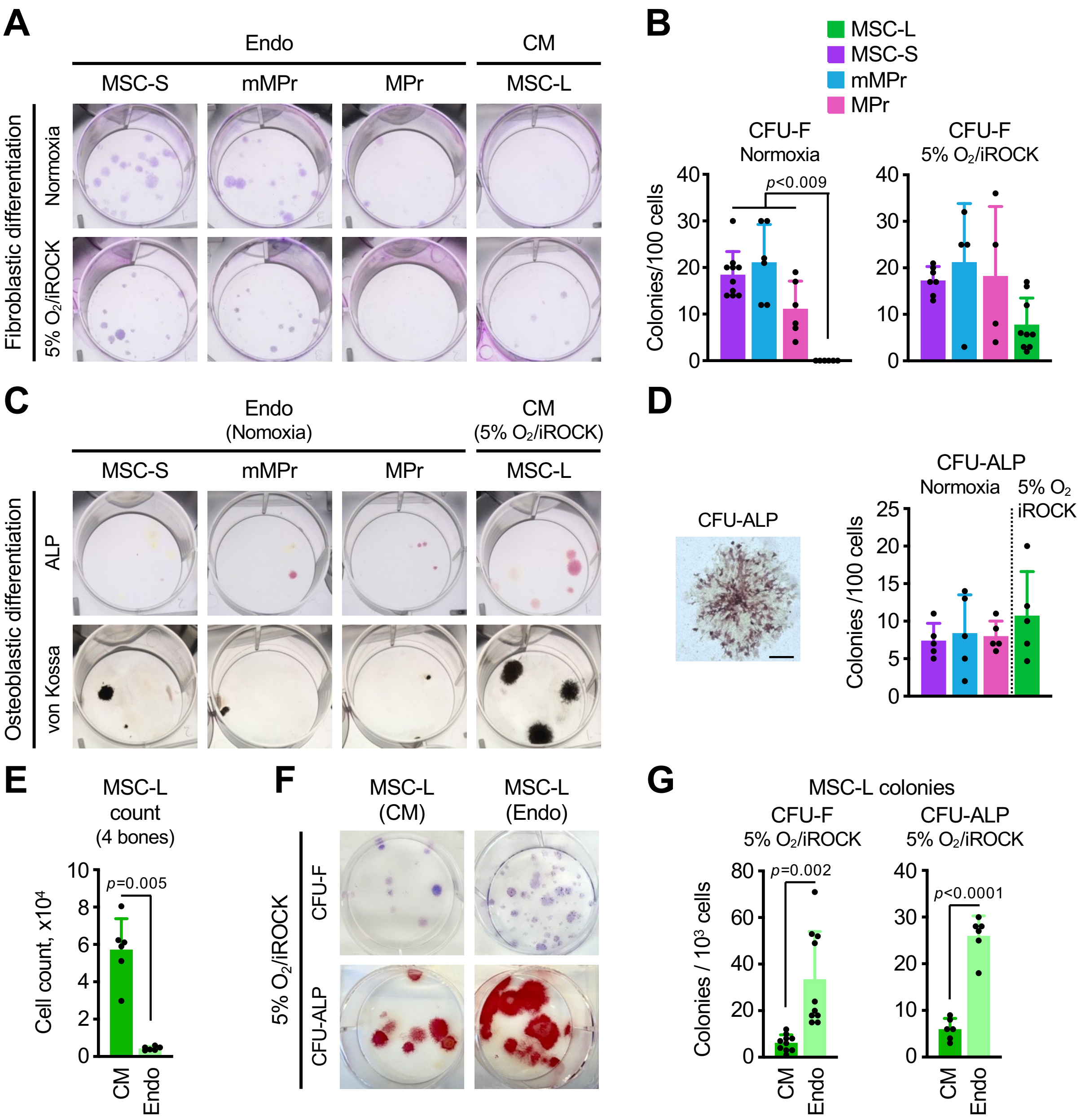
A
Endo
CM
MSC-S
mMPr
MPr
MSC-L
Fibroblastic differentiation
Normoxia
5% O2/iROCK
B
MSC-L
MSC-S
mMPr
MPr
CFU-F
Normoxia
p<0.009
CFU-F
5% O2/iROCK
Colonies/100 cells
C
Endo
(Nomoxia)
CM
(5% O2/iROCK)
MSC-S
mMPr
MPr
MSC-L
Osteoblastic differentiation
ALP
von Kossa
D
CFU-ALP
CFU-ALP
Normoxia
5% O2
iROCK
Colonies /100 cells
E
MSC-L
count
(4 bones)
p=0.005
Cell count, x10^4
CM
Endo
F
MSC-L
(CM)
MSC-L
(Endo)
5% O2/iROCK
CFU-F
CFU-ALP
G
MSC-L colonies
CFU-F
5% O2/iROCK
p=0.002
CFU-ALP
5% O2/iROCK
p<0.0001
Colonies / 10^3 cells
CM
Endo
CM
Endo

Supplemental Figure 10. Functional properties of mesenchymal cell types. A-B, Representative images (A) and quantification (B) of colony forming unit-fibroblast (CFU-F) obtained from the indicated Endo and CM populations. FACS-sorted cells (200 cells/35-mm well) were cultured for 11 days in normoxia or for 8 days in 5% O_2 hypoxia with a ROCK kinase inhibitor (iROCK). **C-D**, Representative images (C) and quantification (D) of alkaline phosphatase expressing colonies (CFU-ALP) and calcifying colonies (von Kossa) obtained after 9 days (CFU-ALP) or 21 days (von Kossa) of induced osteoblastic differentiation of CFU-F colonies. A representative photomicrograph of a CFU-ALP colony (scale bar: 1 mm) is shown on the left in (D). **E**, Quantification of MSC-L numbers in CM (4 plugs) and Endo (4 bones, 2 tibiae + 2 femurs) preparations. **F-G**, Representative images (F) and quantification (G) of CFU-F and CFU-ALP colonies obtained from CM or Endo MSC-Ls. FACS-sorted cells (1,000 cells/35-mm well) were cultured for 7 days in 5% O_2 hypoxia/iROCK conditions for CFU-F scoring and for an additional 14 days in induced osteoblastic differentiation conditions for CFU-ALP scoring. Data in (B,D,E,G) are means ± S.D. with points showing values for individual mice. *P. values* , derived from one-way ANOVA with Tukey's *post hoc* test (B,D), or Welch's t test (E,G).

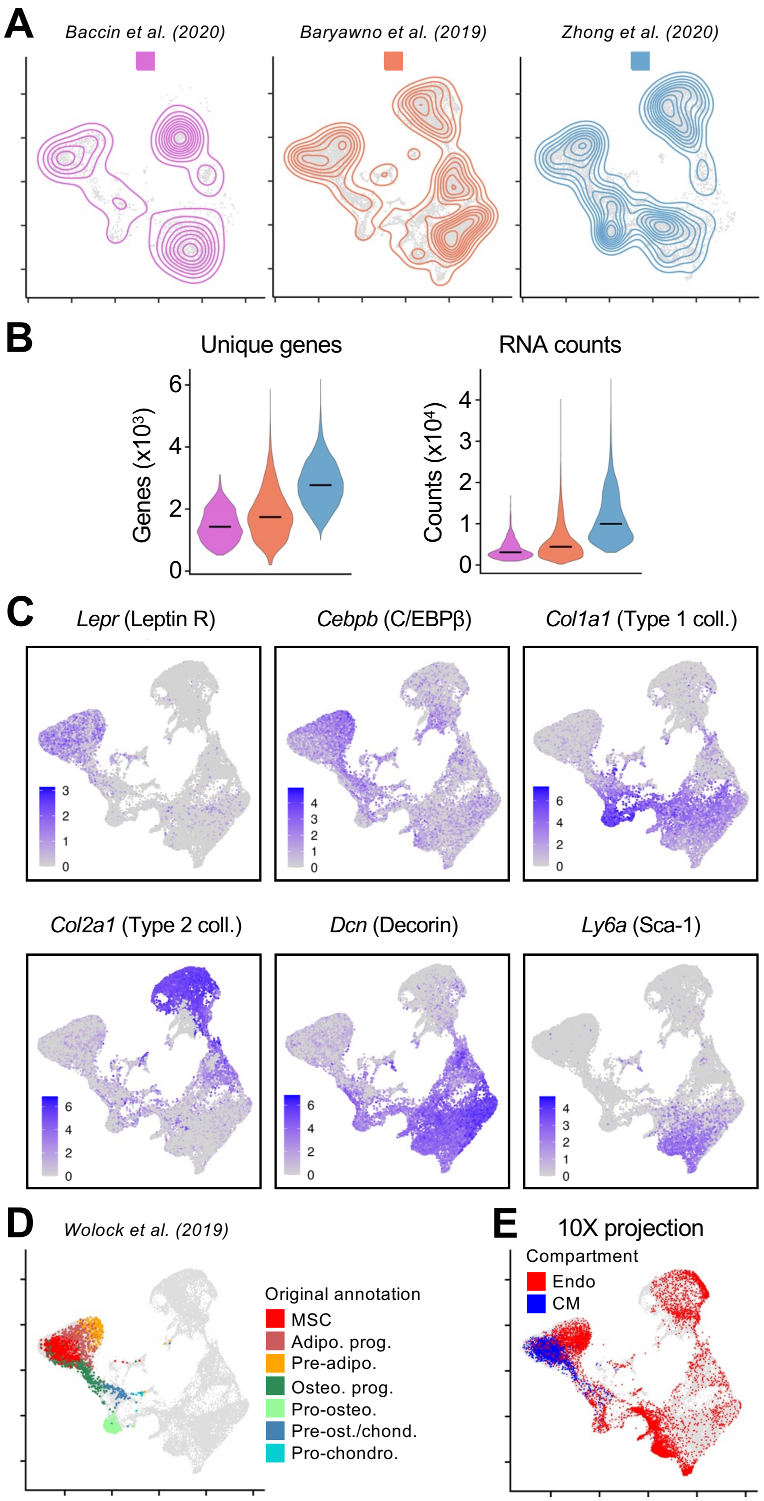
A
Baccin et al. (2020)
Baryawno et al. (2019)
Zhong et al. (2020)
B
Unique genes
Genes (x10³)
RNA counts
Counts (x10⁴)
C
Lepr (Leptin R)
Cebpb (C/EBPβ)
Col1a1 (Type 1 coll.)
Col2a1 (Type 2 coll.)
Dcn (Decorin)
Ly6a (Sca-1)
D
Wolock et al. (2019)
Original annotation
MSC
Adipo. prog.
Pre-adipo.
Osteo. prog.
Pro-osteo.
Pre-ost./chond.
Pro-chondro.
E
10X projection
Compartment
Endo
CM

Supplemental Figure 11. Construction of the 10X mesenchymal atlas. **A**, UMAPs of individual 10X scRNA-seq datasets derived from the indicated previous publications that were integrated to form the composite 10X mesenchymal atlas. **B**, Number of unique genes and gene counts from color-coded individual datasets, with medians. **C**, Feature plots showing expression of indicated genes in the 10X mesenchymal atlas. **D**, Projection of previously annotated 10X stromal cell transcriptomes onto the 10X mesenchymal atlas, with original annotation: adipo.: adipocyte, osteo.: osteoblast; chond.: chondroblast. **E**, Projection of 10X stroma transcriptomes from this study onto the 10X mesenchymal atlas, colored by spatial compartment as CM (blue) and Endo (red).

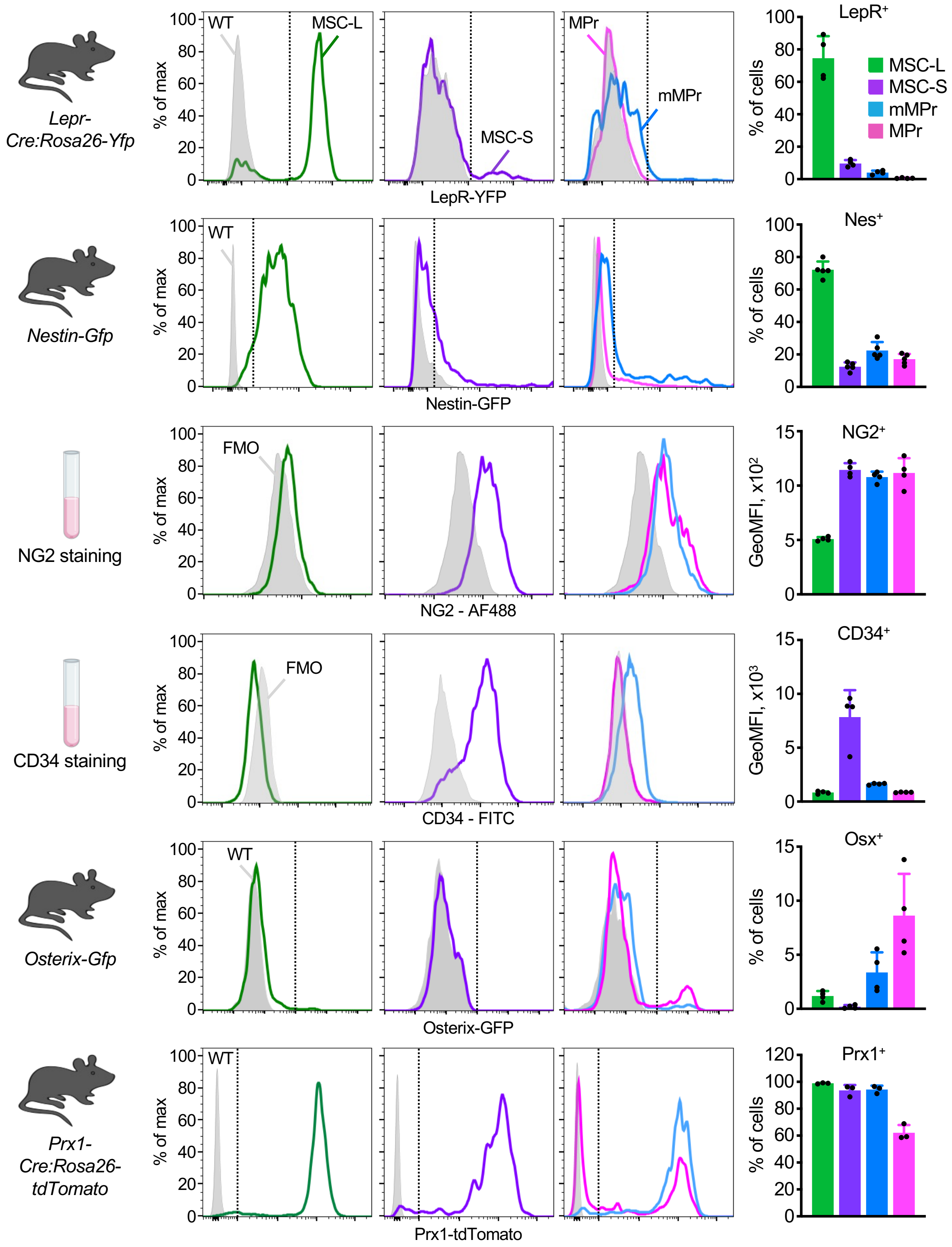
Lepr-Cre:Rosa26-Yfp
Nestin-Gfp
NG2 staining
CD34 staining
Osterix-Gfp
Prx1-Cre:Rosa26-tdTomato
% of max
WT
MSC-L
MSC-S
MPr
mMPr
FMO
LepR-YFP
Nestin-GFP
NG2 - AF488
CD34 - FITC
Osterix-GFP
Prx1-tdTomato
LepR+
Nes+
NG2+
CD34+
Osx+
Prx1+
% of cells
GeoMFI, x10²
GeoMFI, x10³

Supplemental Figure 12 . Expression of fluorescent and protein markers among major stromal cell types. Schematic of the used fluorescent reporter mice or antibody staining (left), representative flow cytometry plots (middle), and quantification of expression in CM MSC-L and Endo MSC-S, mMPr and MPr. Wild type (WT) staining controls are included for the analyses of reporter mice and results are expressed as proportion of cells expressing the fluorescent marker. Fluorescence minus one (FMO) controls are included for the analyses of antibody staining and results are expressed as geometric mean fluorescence intensity (GeoMFI) measurements. Nes: nestin, Osx: osterix. Data are means ± S.D. with points showing values for individual mice.

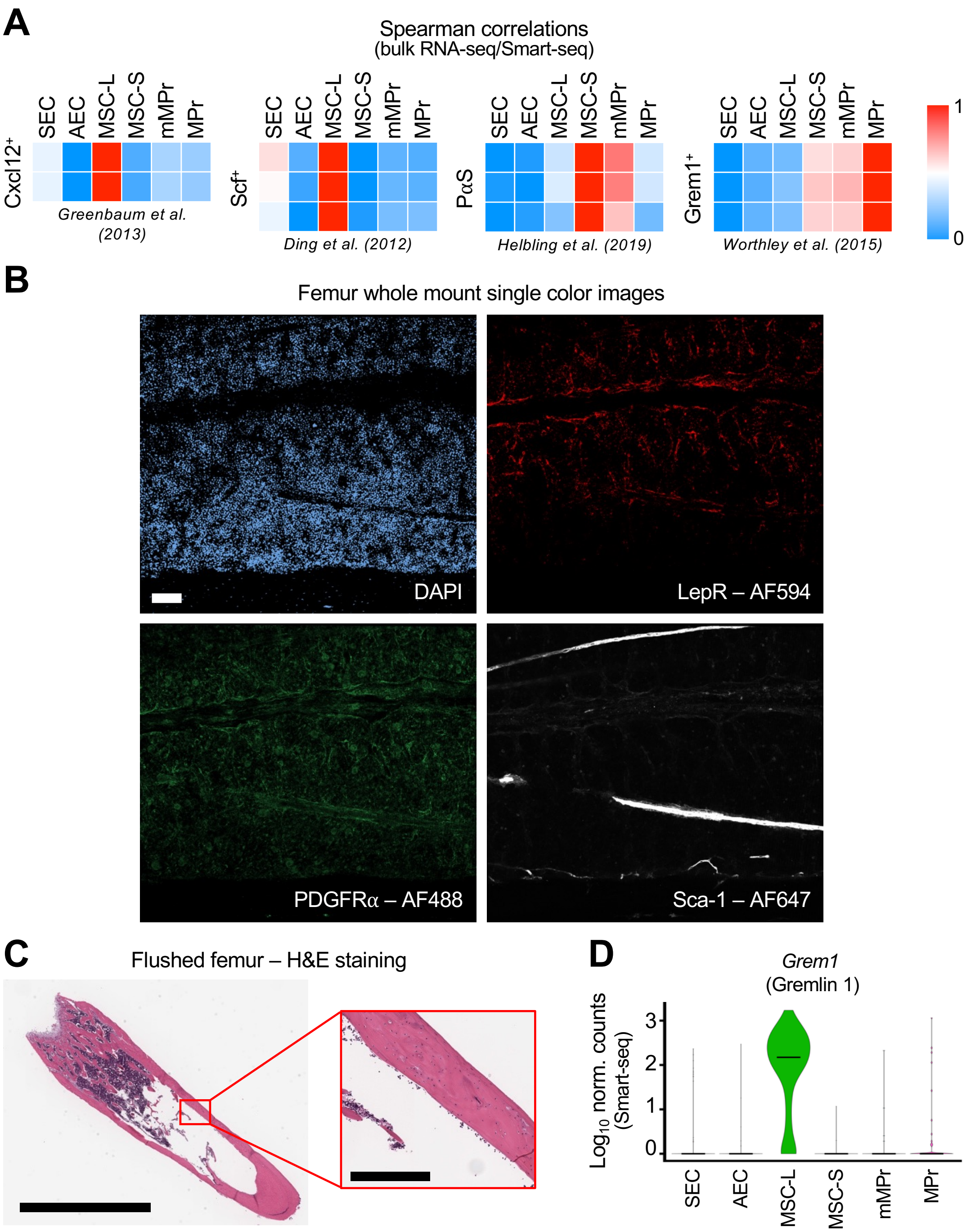
A
Spearman correlations
(bulk RNA-seq/Smart-seq)
SEC
AEC
MSC-L
MSC-S
mMPr
MPr
Cxcl12+
Greenbaum et al. (2013)
Scf+
Ding et al. (2012)
PαS
Helbling et al. (2019)
Grem1+
Worthley et al. (2015)
1
0
B
Femur whole mount single color images
DAPI
LepR – AF594
PDGFRα – AF488
Sca-1 – AF647
C
Flushed femur – H&E staining
D
Grem1
(Gremlin 1)
Log10 norm. counts
(Smart-seq)
0
1
2
3

Supplemental Figure 13. Spatial localization of MSCs. A, Spearman correlation between Smart-seq stromal populations with bulk RNA sequencing data for the indicated cell types obtained from previous publications. PαS: PDGFR-α^+/Sca-1^+ cells. Grem1: Gremlin 1. **B**, Representative single channel confocal image at 10X magnification from femur whole mount preparation, stained with indicated markers. Scale bar = 100 μm. **C**, Femur sectioned and stained with hematoxylin and eosin (H&E) after flushing the marrow plug. Scale bar for low power image = 3 mm. Scale bar for high magnified image = 250 μm. **D**, Differential expression of *Grem1* in purified Smart-seq populations. Results are shown as violin plots of Log10 normalized (norm.) Smart-seq counts with median.

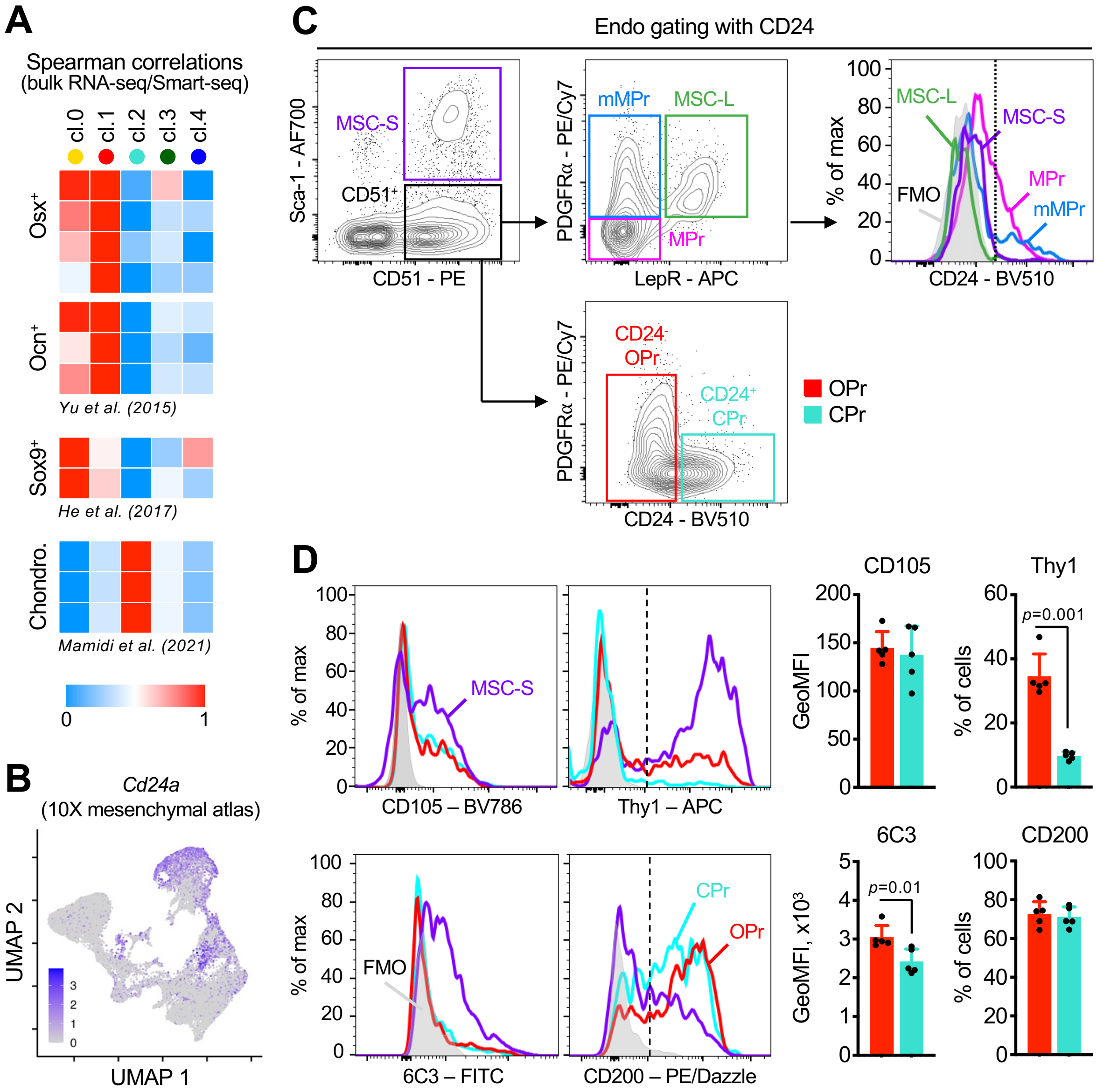
A
Spearman correlations
(bulk RNA-seq/Smart-seq)
cl.0
cl.1
cl.2
cl.3
cl.4
Osx+
Ocn+
Yu et al. (2015)
Sox9+
He et al. (2017)
Chondro.
Mamidi et al. (2021)
0
1
B
Cd24a
(10X mesenchymal atlas)
UMAP 2
UMAP 1
C
Endo gating with CD24
Sca-1 - AF700
MSC-S
CD51+
CD51 - PE
PDGFRα - PE/Cy7
mMPr
MSC-L
MPr
LepR - APC
% of max
MSC-L
MSC-S
FMO
MPr
mMPr
CD24 - BV510
CD24- OPr
CD24+ CPr
CD24 - BV510
OPr
CPr
D
% of max
MSC-S
CD105 – BV786
Thy1 – APC
CD105
GeoMFI
Thy1
p=0.001
% of cells
FMO
6C3 – FITC
CPr
OPr
CD200 – PE/Dazzle
6C3
p=0.01
GeoMFI, x10³
CD200
% of cells

Supplemental Figure 14. Extended characterization of Endo mesenchymal cells. **A**, Spearman correlation between identified Smart-seq PHATE Louvain clusters with bulk RNA sequencing data for the indicated cell types obtained from previous publications. Osx: osterix, Ocn: osteocalcin, Chondro.: primary chondrocytes. **B**, Feature plot showing expression of *Cd24a* in the 10X mesenchymal atlas. **C**, Representative flow cytometry plots showing Endo gating with CD24 for CPr and OPr identification (bottom) and expression of CD24 in major Endo stromal populations (right). D, Representative flow cytometry plots (left) and quantification (right) of expression of the indicated markers in Endo populations. GeoMFI: geometric mean fluorescence intensity. Data are means ± S.D. with points showing values for individual mice. *P. values*, derived from Welch's t test.

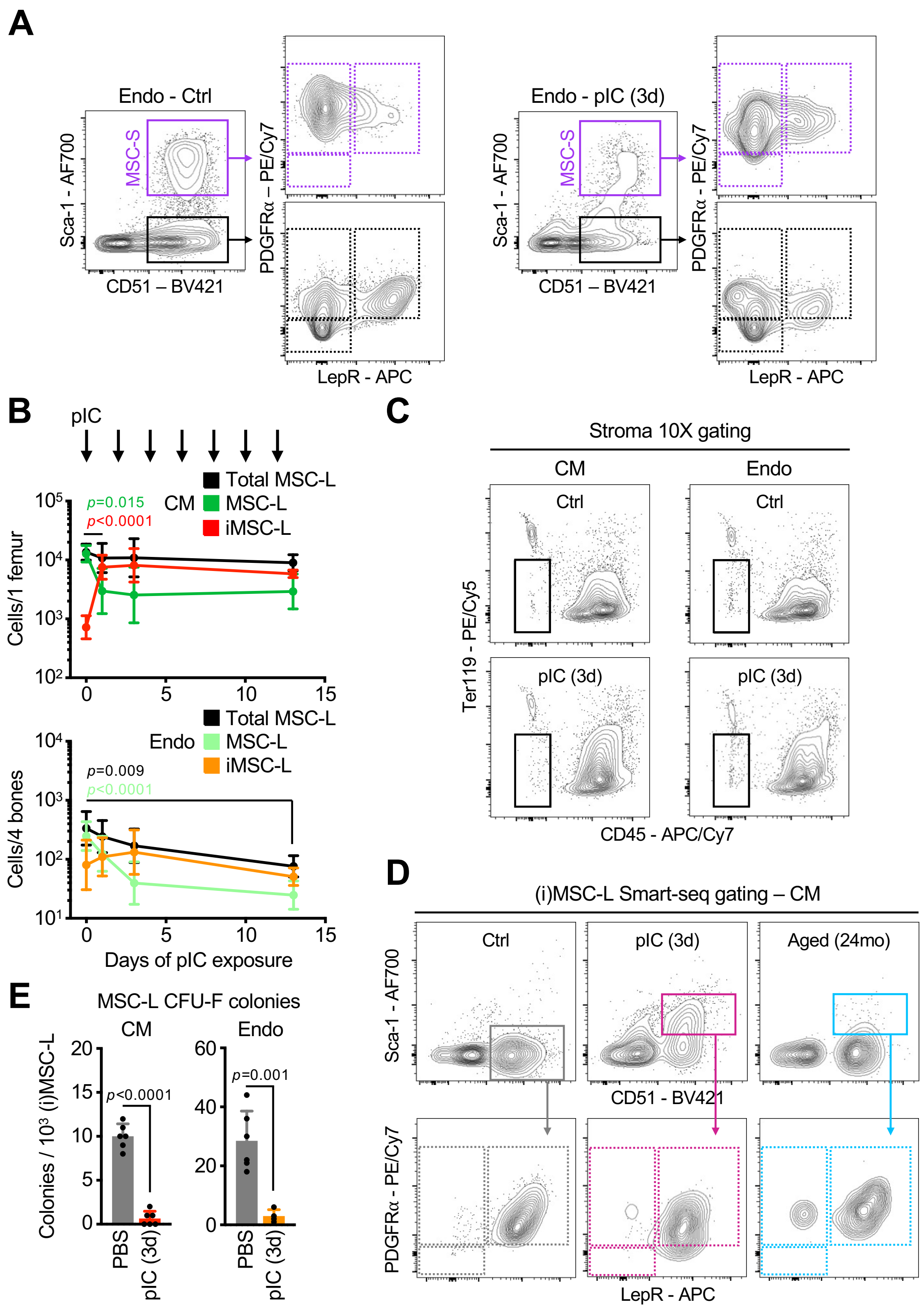
A
Endo - Ctrl
Sca-1 - AF700
MSC-S
CD51 – BV421
PDGFRα – PE/Cy7
LepR - APC
Endo - pIC (3d)
B
pIC
Total MSC-L
CM
MSC-L
iMSC-L
p=0.015
p<0.0001
Cells/1 femur
Endo
Cells/4 bones
p=0.009
p<0.0001
Days of pIC exposure
C
Stroma 10X gating
CM
Endo
Ctrl
pIC (3d)
Ter119 - PE/Cy5
CD45 - APC/Cy7
D
(i)MSC-L Smart-seq gating – CM
Ctrl
pIC (3d)
Aged (24mo)
CD51 - BV421
PDGFRα - PE/Cy7
LepR - APC
E
MSC-L CFU-F colonies
Colonies / 10³ (i)MSC-L
p<0.0001
p=0.001
PBS
pIC (3d)

Supplemental Figure 15. Gating schemes used for isolation of cells for sequencing. A, Representative flow cytometry plots of endosteal (Endo) stromal cells from PBS- (Ctrl) or 3-day-pIC treated mice. **B**, Quantification of MSC-L and iMSC-L in CM (per plug) and Endo (per 4 bones, 2 tibiae + 2 femurs) at indicated times following repeated pIC injections. Total MSC-L represent the sum of MSC-L + iMSC-L at each location. Data are means ± S.D. with n = 3-5 mice per time point. **C-D**, Flow cytometry gating schemes used for isolation of cells for 10X single cell RNA sequencing from PBS and pIC-treated mice (C), or for isolation of single (i)MSC-L cells for Smart-seq (D). **E**, Quantification of CFU-F colonies obtained from CM and Endo MSC-Ls isolated from PBS and pIC-treated mice. Colonies were stained and counted after 7 days. Data are means ± S.D. and points represent individual mice. *P. values*, derived from two-way ANOVA with Sidak's post hoc test (B), or Welch's t test (E).

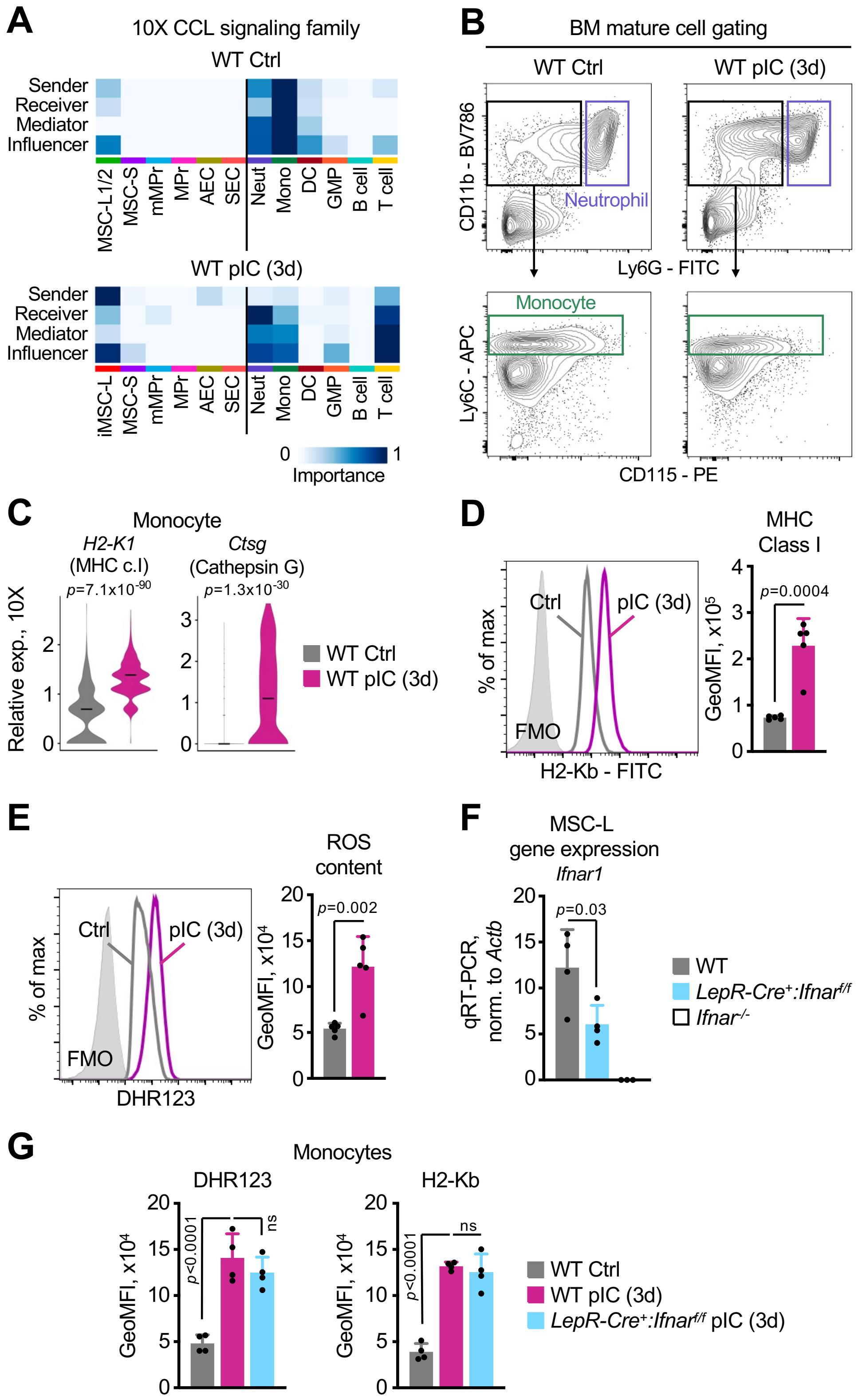
A
10X CCL signaling family
WT Ctrl
Sender
Receiver
Mediator
Influencer
MSC-L1/2
MSC-S
mMPr
MPr
AEC
SEC
Neut
Mono
DC
GMP
B cell
T cell
WT pIC (3d)
iMSC-L
0
1
Importance
B
BM mature cell gating
WT Ctrl
WT pIC (3d)
CD11b - BV786
Neutrophil
Ly6G - FITC
Monocyte
Ly6C - APC
CD115 - PE
C
Monocyte
H2-K1
(MHC c.I)
p=7.1x10-90
Ctsg
(Cathepsin G)
p=1.3x10-30
Relative exp., 10X
WT Ctrl
WT pIC (3d)
D
MHC Class I
% of max
Ctrl
pIC (3d)
FMO
H2-Kb - FITC
p=0.0004
GeoMFI, x10^5
E
ROS content
% of max
Ctrl
pIC (3d)
FMO
DHR123
p=0.002
GeoMFI, x10^4
F
MSC-L gene expression
Ifnar1
p=0.03
qRT-PCR, norm. to Actb
WT
LepR-Cre+:Ifnarf/f
Ifnar-/-
G
Monocytes
DHR123
H2-Kb
GeoMFI, x10^4
p<0.0001
ns
WT Ctrl
WT pIC (3d)
LepR-Cre+:Ifnarf/f pIC (3d)

Supplemental Figure 16. iMSC-L modulate monocyte dynamics in the BM niche. A, Heatmaps showing major participants in CCL family signaling interactions between indicated mature BM and stromal cell types identified by CellChat analyses of 10X scRNA-seq datasets of PBS or 3 day-pIC-injected mice. **B**, Flow cytometry gating scheme for identification of neutrophils and monocytes in BM. **C**, Expression of activation genes in monocytes cluster from 10X BM dataset. Results are shown as violin plots of relative expression (exp.) of 10X SCT transformed counts with median. **D-E**, Representative flow cytometry plots (left) and quantification (right) of MHC class I (H2-Kb) expression (D) and DHR123 expression estimating reactive oxygen species (ROS) content (E) in monocytes of PBS or 3 day-pIC-injected mice, with fluorescence minus one (FMO) control. Results are expressed as geometric mean fluorescence intensity (GeoMFI). **F**, Expression of *Ifnar1* encoding IFNAR1 in MSC-Ls isolated from the indicated mouse genotypes. qRT-PCR results are normalized to *Actb* expression. **G**, Quantification of ROS content (DHR123) and MHC class I expression (H2-Kb) by flow cytometry in monocytes of PBS or 3 day-pIC-injected mice from the indicated genotypes. Data in (D,E,F,G) are means ± S.D. with points showing values for individual mice. *P. value*s, derived from Student's t test (D,E), Wilcoxon rank sum test (C), or one-way ANOVA with Tukey's *post hoc* test (F,G).
